# Quantitative Serial MRI of the Treated Fibroid Uterus

**DOI:** 10.1371/journal.pone.0089809

**Published:** 2014-03-07

**Authors:** Kirsty I. Munro, Michael J. Thrippleton, Alistair R. W. Williams, Graham McKillop, Jane Walker, Andrew W. Horne, David E. Newby, Richard A. Anderson, Scott I. Semple, Ian Marshall, Steff C. Lewis, Robert P. Millar, Mark E. Bastin, Hilary O. D. Critchley

**Affiliations:** 1 MRC Centre for Reproductive Health, University of Edinburgh, Edinburgh, United Kingdom; 2 Centre for Clinical Brain Sciences, University of Edinburgh, Edinburgh, United Kingdom; 3 Royal Infirmary of Edinburgh, NHS Lothian, Edinburgh, United Kingdom; 4 Centre for Cardiovascular Science, University of Edinburgh, Edinburgh, United Kingdom; 5 Clinical Research Imaging Centre, University of Edinburgh, Edinburgh, United Kingdom; 6 Centre for Population Health Sciences, University of Edinburgh, Edinburgh, United Kingdom; 7 Mammal Research Institute, University of Pretoria, Pretoria, South Africa; 8 UCT/MRC Receptor Biology Unit, University of Cape Town, Cape Town, South Africa; 9 Centre for Integrative Physiology, University of Edinburgh, Edinburgh, United Kingdom; Imperial College London, United Kingdom

## Abstract

**Objective:**

There are no long-term medical treatments for uterine fibroids, and non-invasive biomarkers are needed to evaluate novel therapeutic interventions. The aim of this study was to determine whether serial dynamic contrast-enhanced MRI (DCE-MRI) and magnetization transfer MRI (MT-MRI) are able to detect changes that accompany volume reduction in patients administered GnRH analogue drugs, a treatment which is known to reduce fibroid volume and perfusion. Our secondary aim was to determine whether rapid suppression of ovarian activity by combining GnRH agonist and antagonist therapies results in faster volume reduction.

**Methods:**

Forty women were assessed for eligibility at gynaecology clinics in the region, of whom thirty premenopausal women scheduled for hysterectomy due to symptomatic fibroids were randomized to three groups, receiving (1) GnRH agonist (Goserelin), (2) GnRH agonist+GnRH antagonist (Goserelin and Cetrorelix) or (3) no treatment. Patients were monitored by serial structural, DCE-MRI and MT-MRI, as well as by ultrasound and serum oestradiol concentration measurements from enrolment to hysterectomy (approximately 3 months).

**Results:**

A volumetric treatment effect assessed by structural MRI occurred by day 14 of treatment (9% median reduction versus 9% increase in untreated women; P = 0.022) and persisted throughout. Reduced fibroid perfusion and permeability assessed by DCE-MRI occurred later and was demonstrable by 2–3 months (43% median reduction versus 20% increase respectively; P = 0.0093). There was no apparent treatment effect by MT-MRI. Effective suppression of oestradiol was associated with early volume reduction at days 14 (P = 0.041) and 28 (P = 0.0061).

**Conclusion:**

DCE-MRI is sensitive to the vascular changes thought to accompany successful GnRH analogue treatment of uterine fibroids and should be considered for use in future mechanism/efficacy studies of proposed fibroid drug therapies. GnRH antagonist administration does not appear to accelerate volume reduction, though our data do support the role of oestradiol suppression in GnRH analogue treatment of fibroids.

**Trial Registration:**

ClinicalTrials.gov NCT00746031

## Introduction

Uterine fibroids (leiomyomas) are present in up to 70% of women of reproductive age [1], many of whom report significant symptoms [2]. The burden of symptomatic fibroids has a major impact on health care use and costs [3], with a significant number of women in the USA reported to have had a hysterectomy for fibroids [2], costing over $2 billion annually [4].

Surgery is the mainstay of the clinical management of symptomatic fibroids but has inherent risks. Medical therapies for improving heavy menstrual bleeding associated with fibroids do not improve symptoms associated with increased uterine volume. Gonadotrophin-releasing hormone (GnRH) agonists are effective at reducing fibroid size [5] but are only licensed for short-term use. There is therefore an unmet clinical need for pharmacologic agents that alleviate all fibroid symptoms, thereby avoiding or delaying surgery, especially in women wishing to preserve fertility. However, in order to assess potential novel interventions, we need sensitive imaging approaches that can accurately identify changes in tissue volume, vascular properties and composition as surrogate biomarkers of therapeutic efficacy.

Against this background, our study aimed to compare serial magnetic resonance imaging (MRI) approaches in monitoring and predicting response to GnRH analogue, a treatment which is expected to cause reductions in fibroid volume and perfusion. We used T2-weighted (T2W) MRI for estimation of uterine and fibroid volume, dynamic contrast-enhanced MRI (DCE-MRI) for assessment of tissue perfusion and permeability, and magnetization transfer MRI (MT-MRI) to assess changes in fibrosis and macromolecular content. While these approaches have been explored extensively in other organs (e.g. [6], [7]), there has been very limited application of DCE-MRI and MT-MRI in the assessment of the fibroid uterus. Our secondary aim was to assess whether the treatment response depends upon the oestrogenic state of subjects. To this end, some of the treated participants received a GnRH antagonist prior to receiving the agonist, in order to induce hypo-oestrogenism more rapidly during the first 1–2 weeks of the study.

## Materials and Methods

The protocol for this trial and supporting CONSORT checklist are available as supporting information; see Checklist S1 and Protocol S1. Following approval from the Lothian Research and Ethics Committee (*LREC 08/MRE00/30*), premenopausal women scheduled for hysterectomy due to symptomatic fibroids were recruited from gynaecology clinics in NHS Lothian, Scotland from April 2009 until April 2010. The presence of benign fibroids was confirmed histologically on hysterectomy specimens. Participants were required to have at least one non-pedunculated fibroid (diameter ≥2 cm) or multiple small fibroids (total uterine volume ≥200 cm^3^) confirmed by pelvic ultrasound. Patients with contra-indications to MRI or to the study drugs were excluded. Subjects using hormonal preparations or tranexamic acid for symptom control were able to continue taking these throughout the study period. All participants provided written informed consent.

### Study design

This was a prospective randomized open-label blind end-point (PROBE) investigational study (http://www.clinicaltrials.gov; NCT00746031). At screening, menstrual, medical, family and drug histories were taken, and blood pressure, body mass index (BMI) and urine were assessed (including human chorionic gonadotropin to exclude pregnancy) as well as blood clinical haematological and biochemical profiles.

Thirty participants were randomized into one of three groups (n = 10 per group). Treatment allocation was assigned by codes in sealed consecutively numbered opaque envelopes provided by an independent statistician. Group 1 patients received 3 doses of a 3.6 mg subcutaneous implant of the GnRH agonist, (Goserelin) on days 1, 28 and 56 (±3 days); Group 2 patients received 3 doses of a 3 mg subcutaneous injection of the GnRH antagonist (Cetrorelix) on days 1, 4 and 7 (±1 day) followed by 3 doses of a 3.6 mg subcutaneous implant of Goserelin on days 7, 35 and 63 (±3 days); Group 3 received no treatment. Day 1 occurred within the first 5 days of the menstrual cycle. Hysterectomy was performed 21–28 days from last dose of GnRH agonist or on day 77–84 for untreated subjects. Neither subjects nor clinical personnel were blinded to treatment allocation. Throughout the study, participants were monitored for adverse events with standard laboratory safety measures.

### Magnetic Resonance Imaging

MRI was acquired using a Siemens Avanto 1.5 tesla clinical scanner using body matrix and spine coil elements (receive) and a body coil (transmit). Scans were performed at baseline, on day 14 (±3 days), on day 28 (±3 days) and within 10 days of scheduled hysterectomy. To reduce bowel motion artefact, subjects received intravenous (oral when venous access was not available) hyoscine 20 mg (Buscopan; Boehringer Ingelheim, Germany) prior to scanning.

**Figure 1:**
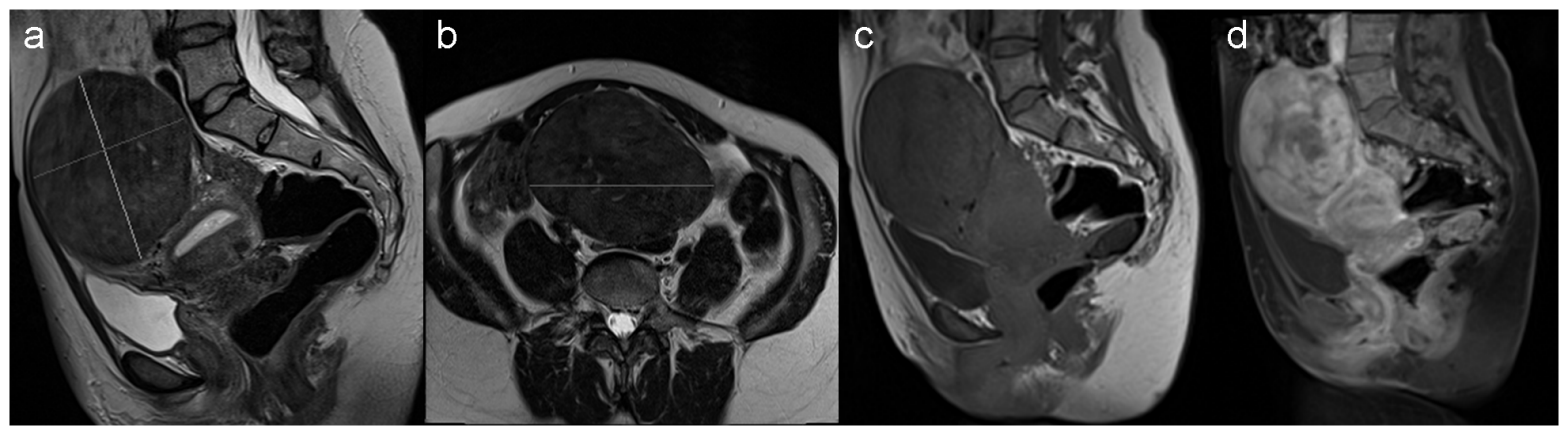
Sagittal (a) and axial-oblique (b) T2-weighted images showing dimensions of a large fibroid. (c) Magnetization transfer-weighted and (d) dynamic contrast-enhanced MRI (DCE-MRI) image (acquired one minute after injection of contrast) of the same patient.

T2W MRI scans (TR/TE = 4470–8480/84–104 ms, slice thickness/spacing = 4/5 mm, field of view (FoV) = 250×250 mm, matrix size = 320×320, 2 signal averages) were acquired in the sagittal and axial-oblique (orthogonal to the uterine lumen) planes (Figure 1a–b) using a fast spin-echo (FSE) pulse sequence. Anterior saturation was applied to reduce respiratory motion artefact and the number of slices was adjusted to obtain full coverage of the uterus.

MT-MRI was acquired in the sagittal plane using a FSE sequence (TR/TE = 797/11 ms, 44 contiguous 3 mm slices, FoV = 300×300 mm, matrix size = 192×192, 2 signal averages (Figure 1c). Magnetization transfer pulses with bandwidth 250 Hz, flip angle 500° and duration 7680 µs were applied with a 1500 Hz-offset from the water resonance frequency. An identical scan was performed without MT pulses.

DCE-MRI with fat suppression (Figure 1d) was acquired in the sagittal plane using a three-dimensional T1-weighted spoiled gradient echo sequence (44 contiguous 3 mm slices, TR/TE = 4.98/1.85 ms, flip angle = 10°, FoV = 300×300 mm, matrix size = 192×192). After the baseline scan, 15 mL gadoteric acid (0.5 mmol/mL; DOTAREM, Guerbet, France) was injected intravenously (Medrad Spectris Solaris EP; http://www.medrad.com) at 3 mL/s and flushed with 0.9% saline. Starting from the time of injection, images were acquired at 15 s intervals; after a total of 12 acquisitions, the interval was increased to 75 s. Pre-contrast T2W FSE scans (TR/TE = 8410/84 ms) with the same slice locations as DCE-MRI were acquired to facilitate accurate ROI placement.

### Image analysis

*Uterine and largest fibroid volumes* were the primary outcomes of the study. To estimate uterine volume, measurements were taken from sagittal T2W images from the internal cervical os to the most distant point of the uterus, and of the longest axis perpendicular to this; a third left-right measurement was made using axial-oblique images. To estimate fibroid volume, orthogonal long- and short-axis measurements of the largest fibroid were taken in the sagittal view (Figure 1a), together with a left-right measurement in the axial-oblique view (Figure 1b). For both structures, the three orthogonal distances *L_i_* were combined using the equation for the volume of an ellipsoid (*V* = π/6×*L*_1_×*L*_2_×*L*_3_). Measurements were made by consensus of two readers (KM and MT) and verified by an experienced radiologist (GM).

#### DCE-MRI

Regions of interest (ROIs) encompassing the largest fibroid were drawn on sagittal T2W images and used to generate averaged DCE-MRI signal intensity curves. For assessment of contrast uptake in the myometrium at baseline, three circular ROIs (diameter ≤8 mm) were drawn in a region of normal-appearing myometrium and the signals from these were combined to generate averaged signal intensity curves. All curves were fitted using the kinetic model of Brix and co-workers [8], [9] using software written in Matlab (MathWorks, Inc., Natick, MA, USA).
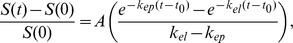
where *S*(0) and *S*(*t*) represent the signal intensity at baseline and time *t* respectively, *A* is a constant of proportionality, *t*_0_ accounts for the unknown arrival time of the contrast agent in the tissue, *k*_el_ is the rate constant for the removal of contrast and *k*_ep_ is the rate constant for transfer of contrast from the extracellular extravascular space to the blood pool. Example data with fitting to this model are shown in Figure S1.

#### MT-MRI

The fibroid tissue ROIs described above were transferred to the MT-MRI images and averaged magnetization transfer ratio (MTR) values were calculated as 100×(*S*_−_−*S*_+_)/*S*_−_, where *S*_+_ and *S*_−_ are the ROI-averaged signal intensities in the MT-weighted and non-MT-weighted images respectively.

### Ovarian activity

Blood samples for circulating serum oestradiol concentrations were collected on days 1, 4, 7 and 11±1 day, at imaging visits ±3 days and on the day of hysterectomy. Serum oestradiol was measured using the ARCHITECT assay (Abbott Diagnostics, Maidenhead, UK) that utilises Chemiluminescent Microparticle Immunoassay (CMIA) technology; the analytical sensitivity was ≤10 pg/mL.

### Sonographic assessment

Ultrasound was performed at the screening visit to identify the location and size of the largest fibroid and subsequently on the same dates as MRI ±3 days. All ultrasound scans were performed transabdominally and, in most cases, transvaginally; volumes were calculated using the latter where image quality permitted. Three measurements of the uterus and largest fibroid were recorded (longitudinal, transverse and anterior-posterior) and volumes were calculated using the ellipsoid equation as above. The position of the largest fibroid was noted to ensure assessment of the same fibroid at each visit. Colour Doppler imaging was carried out at all visits, with impedance to uterine blood flow distal to the point of sampling estimated using the pulsatility index (PI), defined as systolic minus end diastolic peak velocity divided by time-averaged maximum velocity [10]. Three measurements were taken from each of the left and right uterine arteries; the mean value was calculated for each side and the two sides were then averaged. All scans were performed using the same Siemens Antares machine. The majority of the scans (79%) were carried out by one radiologist (JW), with the remainder performed by a second radiologist (TF).

### Statistical methods

MRI, hormone and ultrasound parameters were compared using the Mann-Whitney *U*-test, while correlations between continuous variables were assessed by Spearman rank correlation analysis. Variables were considered statistically significant using two-sided P<0.05. Treatment response by structural, DCE-MRI and MT-MRI was assessed at each time point using the first (pre-treatment) scan as the baseline. This was a hypothesis-generating study, so no formal adjustment for multiple testing has been used.

## Results

### Patient demographics and recruitment

**Figure 2:**
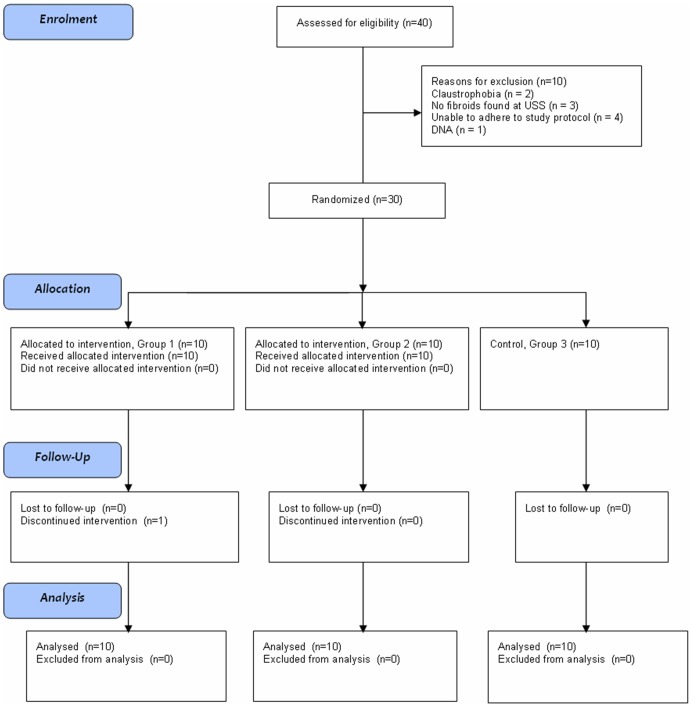
CONSORT flow diagram showing enrolment and progress of study participants.

Forty patients were assessed for participation, of whom nine did not meet the inclusion criteria and one did not attend the screening appointment. The remaining thirty women were randomized into one of three groups and all completed the study to the endpoint of hysterectomy (Figure 2). One patient in Group 1 declined the third dose of GnRH agonist due to problematic heavy menstrual bleeding. MRI scans took place on days −25 to +1 (median −6, interquartile range 8), days 13 to 18 (median 15, interquartile range 2), days 25 to 33 (median 29, interquartile range 1) and days 48 to 100 (median 77, interquartile range 3) respectively.

The groups were well matched for age, ethnic origin, parity and BMI (Table 1). No patient developed withdrawal criteria during the study, nor received the incorrect treatment. Adverse events are described in (Text S1 and Table S1). This was a hypothesis-generating study: adjustment for multiple testing was not used for calculating the exploratory P-values presented below.

**Table 1:** Patient demographics: race, age, parity and BMI distribution across the treatment groups.

		Treatment Group			
Variable		Group 1 (n = 10)	Group 2 (n = 10)	Group 3 (n = 10)	All subjects (n = 30)
Race, n (%)	Black	1 (10)	0 (0)	0 (0)	1 (3.3)
	Caucasian	9 (90)	10 (100)	10 (100)	29 (96.7)
Age, years	Mean	45.4	45.9	46	45.8
	Min, Max	40, 52	42, 53	42, 52	40, 53
Parity, n (%)	Nulliparous	3 (30)	5 (50)	4 (40)	12 (40)
	Parous	7 (70)	5 (50)	6 (60)	18 (60)
BMI, kg/m^2^	Mean	30.1	28.6	32.0	30.2
	Min, Max	19, 43	21, 39.5	22.1, 37.2	19, 43

### Ovarian activity

**Figure 3:**
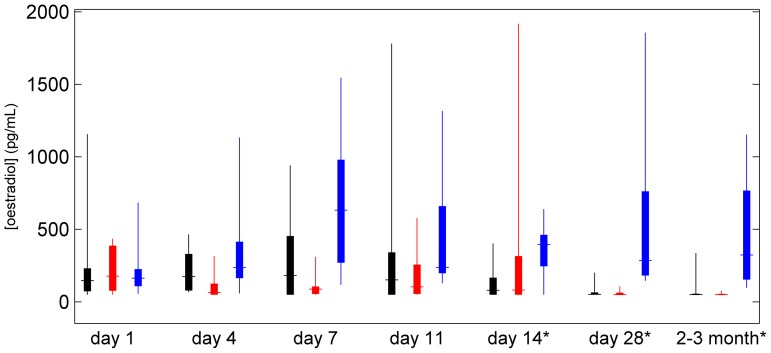
Box and whisker plot showing blood oestradiol concentration, with groups 1, 2 and 3 displayed in black, red and blue respectively. * indicates samples taken on MRI scan days.

Serum oestradiol concentrations for the three groups are shown in Figure 3. At baseline (day 1), the concentrations in the groups were similar. At day 4, serum oestradiol concentrations were raised in Group 1 compared with Group 2 (*P* = 0.040), consistent with suppression of the oestradiol “flare” by the GnRH antagonist. However, no subsequent differences in serum oestradiol concentrations were observed between the two treated groups, indicating little difference in ovarian activity. Since the treatment regimes of Groups 1 and 2 were similar (GnRH agonist only) after the first week, these subjects were combined into a single treatment group for analysis of imaging data. Treated subjects displayed reduced serum oestradiol concentrations relative to untreated women at days 7 (*P* = 0.0018), 11 (*P* = 0.052), and at second (day 14; *P* = 0.023), third (day 28; *P*<0.001) and final (2–3 months; *P*<0.001) imaging visits.

### Volume (T2W-MRI and ultrasound)

**Figure 4:**
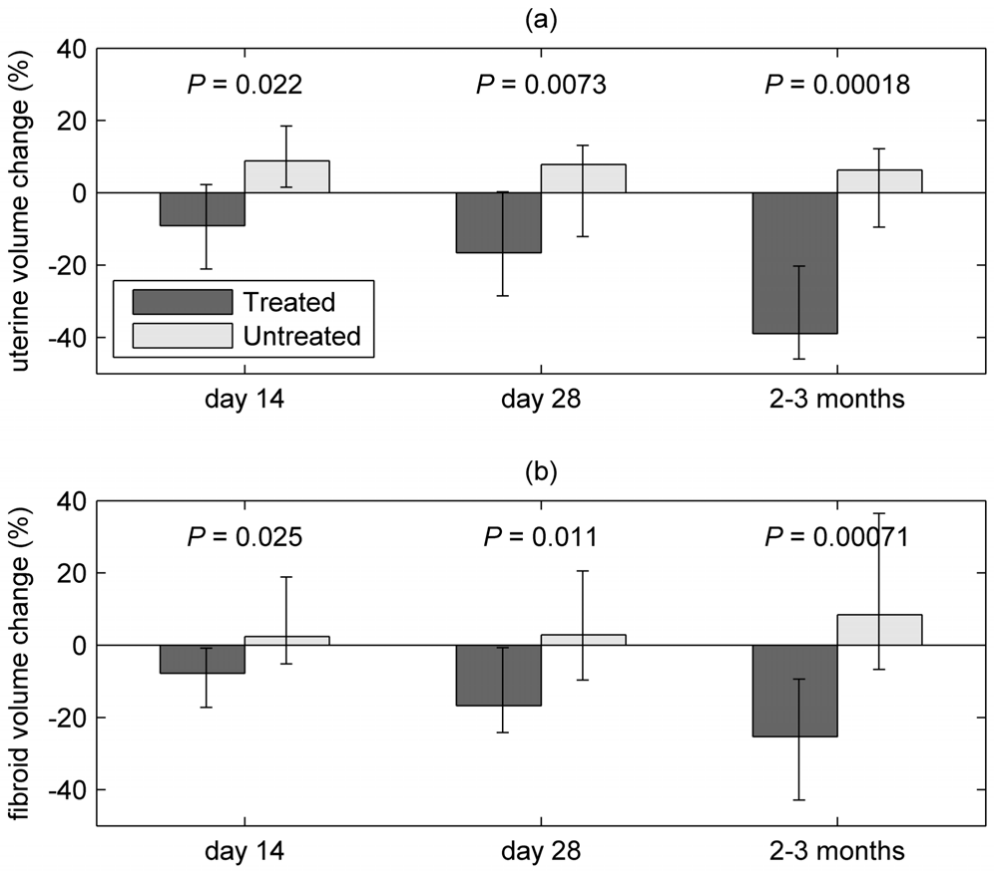
Median % volume change of (a) uterus and (b) largest fibroid from baseline at day 14, day 28 and 2 to 3 months (i.e. within 10 days of hysterectomy), measured by T2-weighted MRI; error bars show the interquartile range. Figure shows data for treated (groups 1 and 2) and untreated participants.

Baseline volumes (Table 2) measured by MRI were similar among treated and untreated participants. Reductions in uterine and fibroid volume among all treated patients (groups 1 and 2 combined) compared with untreated patients (group 3) were observed at day 14, and the size of this treatment effect increased throughout the study. Median reductions in uterine (fibroid) volume among treated patients were 9.1 (7.7) %, 16.6 (16.7) % and 39.0 (25.3) % at day 14, day 28 and 2–3 months respectively (Figure 4, Table 2); there were no substantial changes in the fibroid and uterine volumes for the untreated group, and the response of groups 1 and 2 did not differ significantly at any of the time points. Initial volume was not associated with final percentage volume change in patients receiving treatment for either uterus (*r_s_* = 0.093, *P* = 0.70) or fibroid (*r_s_* = −0.12, *P* = 0.62). Ultrasound measurements showed similar volume changes (Figure S2) but a significant treatment effect was not identifiable until the final measurement.

**Table 2:** MRI parameters at baseline and follow-up scans: uterine and fibroid volume, *k*_ep_ (DCE-MRI) and magnetization transfer ratio.

		Treated				Untreated			
Variable		Baseline	day 14	day 28	2–3 months	Baseline	day 14	day 28	2–3 months
uterine volume (ml)	median	555.2	440.5	468.0	351.1	556.9	478.5	484.3	500.8
	min	119.4	83.8	80.6	55.7	201.1	233.0	176.8	261.3
	max	1990.3	1885.5	1367.7	1326.5	1003.7	1019.6	1114.6	1044.1
fibroid volume (ml)	median	249.6	223.3	181.4	145.7	127.7	141.7	141.2	131.9
	min	2.4	1.9	2.4	1.8	29.3	34.0	23.3	41.1
	max	1179.6	1053.5	713.5	739.8	490.7	723.2	591.6	669.8
DCE-MRI (*k*_ep_/s^−1^)	median	0.0854	0.0507	0.0591	0.0389	0.0492	0.0734	0.0748	0.0931
	min	0.0201	0.0241	0.0153	0.0169	0.0279	0.0242	0.0160	0.0275
	max	0.2123	0.1818	0.1956	0.1364	0.1529	0.1028	0.1684	0.1220
MTR (%)	median	12.0	11.7	13.0	12.4	12.2	11.3	12.8	12.2
	min	10.1	6.8	9.7	7.5	6.6	5.7	5.0	6.1
	max	20.3	18.5	15.9	18.9	15.3	15.4	17.1	16.1

### Perfusion and permeability (DCE-MRI and Doppler Ultrasound)

**Figure 5:**
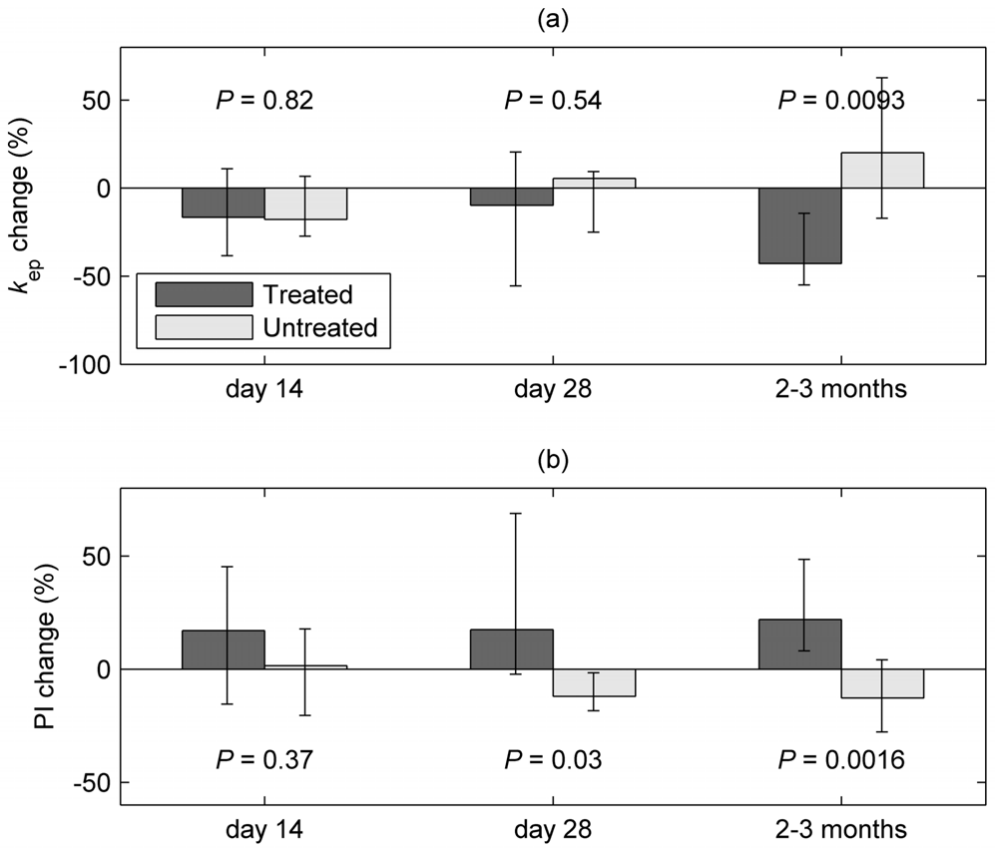
Changes in uterine vascular properties at day 14, day 28 and 2 to 3 months (i.e. within 10 days of hysterectomy). (a) Shows median % change in the fibroid dynamic contrast-enhanced MRI (DCE-MRI) parameter *k*_ep_ from baseline, while (b) shows corresponding changes in pulsatility index (PI) as measured by Doppler ultrasound of the uterine arteries. Error bars show the interquartile range. Figure shows data for treated (groups 1 and 2) and untreated participants.

Changes in the contrast uptake properties of the largest fibroid (assessed by DCE-MRI parameter *k*_ep_) are shown in Figure 5 and Table 2. In agreement with the observed volume changes, a reduction was seen among the combined treated patients (groups 1 and 2) relative to untreated patients (group 3). However, this was apparent only at the final measurement (2–3 months), with a 42.7% median reduction in treated patients compared with a 20.1% increase for untreated participants (*P* = 0.0093). In contrast to volumetric assessment, no treatment effect was observed at earlier measurements. The treatment response for groups 1 and 2 did not differ significantly at any of the follow-up scans. Baseline myometrium (though not fibroid) *k*_ep_ was associated with final uterine volume change for treated patients (*r_s_* = −0.57; *P* = 0.0099). Myometrium (though not fibroid) *k*_ep_ was also negatively associated with uterine (*r*_s_ = −0.38; *P* = 0.049) and fibroid (*r_s_* = −0.51; *P* = 0.0063) volume among all patients at baseline.

Doppler ultrasound assessment of the uterine arteries (Figure 5b) showed complementary increases in PI in treated versus untreated patients at day 28 (*P* = 0.030) and at 2–3 months (*P* = 0.0016). However, pre-treatment Doppler ultrasound did not predict final uterine volume change in treated patients (*r_s_* = 0.10, *P* = 0.67).

### Fibroid composition (MT-MRI)

In contrast to the observed volumetric response, serial MT-MRI revealed no differences in response between treated and untreated patients (or between the two treated groups) at any of the follow-up scans (Table 2, Table S2). Baseline MTR was not associated with final uterine volume change, although baseline MTR was negatively associated with initial uterine (*r*_s_ = −0.46; *P* = 0.011) and fibroid (*r*_s_ = −0.52; *P* = 0.0036) volume.

### Hypo-oestrogenism and treatment effect

**Figure 6:**
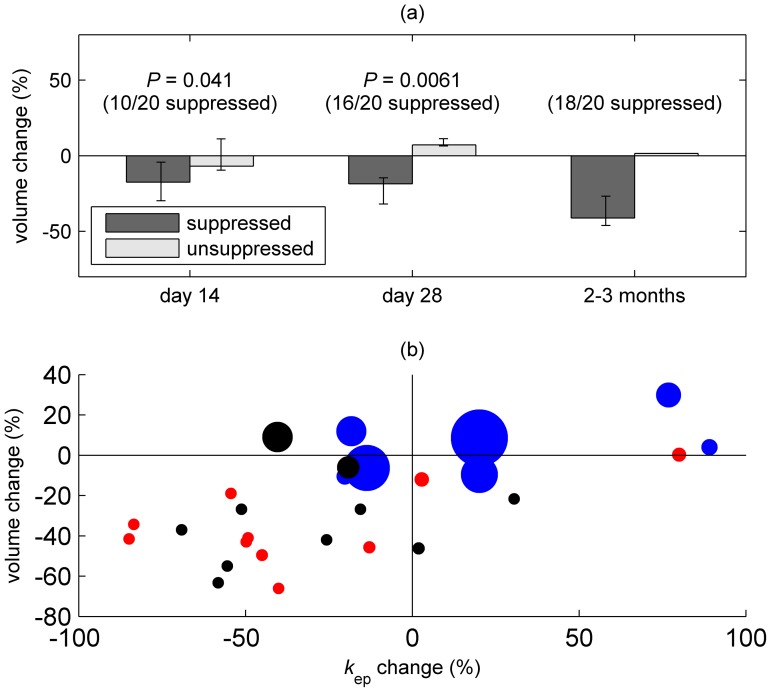
Relationship between oestradiol concentration, and volumetric and vascular changes. (a) shows median uterine volume change, assessed by T2-weighted MRI; error bars show the interquartile range. Data are shown for treated participants (groups 1 and 2), classified as oestradiol-suppressed and -unsuppressed as described in the text. (b) shows changes in uterine volume and fibroid *k*_ep_ (DCE-MRI) for all patients (group 1: black, group 2: red, group 3: blue) at the final MRI scan. The areas of the data points are proportional to blood oestradiol concentration at the same time point (the largest data point corresponds to a value of 1153 pg/mL).

As discussed above, there were no differences in serum oestradiol concentrations after day 4 between those patients receiving an initial regime of GnRH antagonist followed by GnRH agonist, and those receiving only GnRH agonist. Consistent with this, we found no differences between these two groups by serial MRI. Nevertheless, there was variation in residual serum oestradiol concentrations among treated patients. To assess the dependence of treatment response on hypo-oestrogenism, treated patients were dichotomised as oestradiol-suppressed ([E2]≤81.5 pg/mL) or unsuppressed ([E2]>81.5 pg/mL), with the median serum oestradiol concentration at day 14 used as the cut-off. As shown in Figure 6a, volume reduction in “suppressed” patients was greater than in “unsuppressed” patients at days 14 and 28 (statistical testing is not reported for the final scan as all but two treated participants were oestradiol-suppressed by this time point). Suppressed patients also showed greater reduction in *k*_ep_ (Figure S3) although the differences did not reach statistical significance; however, as illustrated in Figure 6b, volume reduction was accompanied in most patients by hypo-oestrogenism and a reduction in *k*_ep_.

## Discussion

The main objective of this study was to compare structural MRI, DCE-MRI and MT-MRI as potential biomarkers of response to fibroid drug treatment. We found that structural MRI was able to identify a significant volumetric treatment effect as early as day 14 following commencement of treatment, and at the following two scans. This effect was detected by standard clinical ultrasound only 2–3 months after commencement of treatment. These data support the use of structural MRI as the preferred technique for detecting early volumetric treatment response and, as far as we are aware, represent the earliest detected volumetric reduction of a GnRH analogue treatment. These data also show for the first time that the change in fibroid volume with GnRH analogue treatment is rapid. The magnitude of the volume changes were comparable to those found in previous studies utilising GnRH agonist therapy [11], [12]. Just as fibroid growth rates have been shown not to be influenced by tumour size [13], we found that fibroid and uterine size did not predict final percentage volume change in treated patients.

### Perfusion and permeability

Assessment of fibroid change in response to treatment by DCE-MRI showed a treatment effect at 2–3 months, complementary to but much later than volumetric changes detected by structural MRI. Treatment may reduce the uterine blood supply in the early stages (as demonstrated by our Doppler ultrasound findings and those of previous investigators [14]), but without affecting the fibroid ‘core’ until later in the treatment cycle, when uterine blood flow has fallen sufficiently to limit the uptake of contrast. Since we assessed averaged contrast uptake across the whole fibroid, such local variations in the treatment effect may be obscured. Furthermore, caution should be exercised in interpreting changes in contrast uptake, which is influenced by multiple properties including blood flow, vascular permeability and the extracellular volume. Shimada *et al* have previously demonstrated a positive association between fibroid vessel density and MRI contrast enhancement [15]. Further work using DCE-MRI with higher temporal resolution and arterial concentration measurement should allow some of these factors to be disentangled [16].

Pre-treatment DCE-MRI of myometrium was negatively associated with the final uterine volume change. It is unclear whether this reflects greater availability of the pharmacologic agent, or simply that a highly perfused uterus is more strongly affected by reductions in blood supply. A previous study using qualitative contrast-enhanced MRI has suggested that enhancing fibroids show greater volume reduction than unenhanced fibroids [17]. Whilst this may reflect a reduced treatment response in poorly-enhanced hyalinised fibroids [18], [19], we did not find pre-treatment fibroid *k*_ep_ to be associated with final volume change.

Qualitative contrast-enhanced MRI is used clinically to assess suitability for uterine artery embolisation treatment and to demonstrate the consequent reductions in perfusion [20]. As far as we are aware, this is the first study in which quantitative DCE-MRI has been employed in patients with fibroids who have been administered GnRH agonist therapy. We have shown that DCE-MRI is sensitive to therapy-induced vascular changes in fibroid tissue and as such it may be useful in future studies of therapeutic efficacy and mechanism. Nevertheless, it will be necessary to balance the acquisition of additional scientific information against the cost of administering contrast agent and increasing the total duration of the imaging protocol.

### Fibroid composition (MT-MRI)

Whilst MT-MRI has been reported in relation to other uterine pathology [21], MT-MRI has not previously been used to assess uterine fibroids, except as an incidental finding during whole body MRI [22]. Here, we were unable to identify a treatment effect by MT-MRI during the study. A possible reason is that GnRH therapy does not substantially alter the fibroid tissue structure on a 2–3 month timescale. Moreover, long acquisition times at 1.5T limited the accuracy of MTR measurement in the presence of patient motion. We have recently piloted faster MT-MRI acquisition at 3T and this permits rapid voxel-based MTR mapping that may enhance the sensitivity of this technique.

### Dependence on hypo-oestrogenism

As expected, initial treatment with GnRH antagonist preceding GnRH agonist therapy resulted in lower serum oestradiol concentrations on day 4. We found greater uterine volume reduction in treated patients with the lowest serum oestradiol concentrations as early as day 14, implying that rapid suppression of ovarian activity is related to early volume reduction. Similar effects were seen by DCE-MRI. It has previously been demonstrated that uterine fibroids are hormone-dependent, with reductions in oestradiol (e.g. following the menopause) causing fibroids to decrease in size [23], [24]. While the underlying mechanism of GnRH analogue-induced fibroid reduction is not well-understood [25], recent work by Khan *et al* [26] found that GnRH agonists reduced the inflammatory reaction, angiogenesis and micro-vessel density and induced apoptosis in uterine fibroids. Our data support the hypothesis that shrinkage is caused by suppression of ovarian activity and mediated by a reduction in perfusion.

### Conclusions

These data demonstrate that structural MRI can detect small, early responses to treatment prior to detection by standard clinical ultrasound. DCE-MRI allows the measurement of a vascular treatment response in fibroid tissue complementary to structural changes following 2–3 months of treatment and may also be a predictor of treatment response. Our data would suggest that MRI should be considered an important imaging modality for assessment of response to novel therapeutic interventions for this important and potentially debilitating condition.

## Supporting Information

Figure S1 **Example DCE-MRI data showing signal enhancement vs. time with fitting to the kinetic model described in the** **Materials and Methods** **section.** Data from four serial scans from the same treated patient are displayed, showing reduction in the perfusion and permeability parameter *k*_ep_.
(TIF)

Figure S2 **Median % volume change of (a) uterus and (b) largest fibroid from baseline at day 14, day 28 and 2 to 3 months (i.e. within 10 days of hysterectomy), measured by ultrasound; error bars show the interquartile range.** Figure shows data for treated and untreated participants.
(TIF)

Figure S3 **Median** ***k*** **_ep_ change, assessed by DCE-MRI; error bars show the interquartile range.** Figure shows data for treated participants, classified as oestradiol-suppressed and -unsuppressed as described in the text.
(TIF)

Table S1 Adverse Events Log (*suspected unexpected serious adverse reaction (SUSAR); reported to MHRA as per protocol).
(DOC)

Table S2 **Absolute median MTR change (%) from baseline.**
(DOC)

Text S1 **Description of Adverse Events and Missing Data.**
(DOC)

Protocol S1 **Trial protocol.**
(DOC)

Checklist S1 **CONSORT Checklist.**
(DOC)
