# Supplementary figures and images for: Quantitative Serial MRI of the Treated Fibroid Uterus
Source: PLoS One. 2014 Mar 7;9(3):e89809. doi: 10.1371/journal.pone.0089809 (PMC3946427; doi:10.1371/journal.pone.0089809)

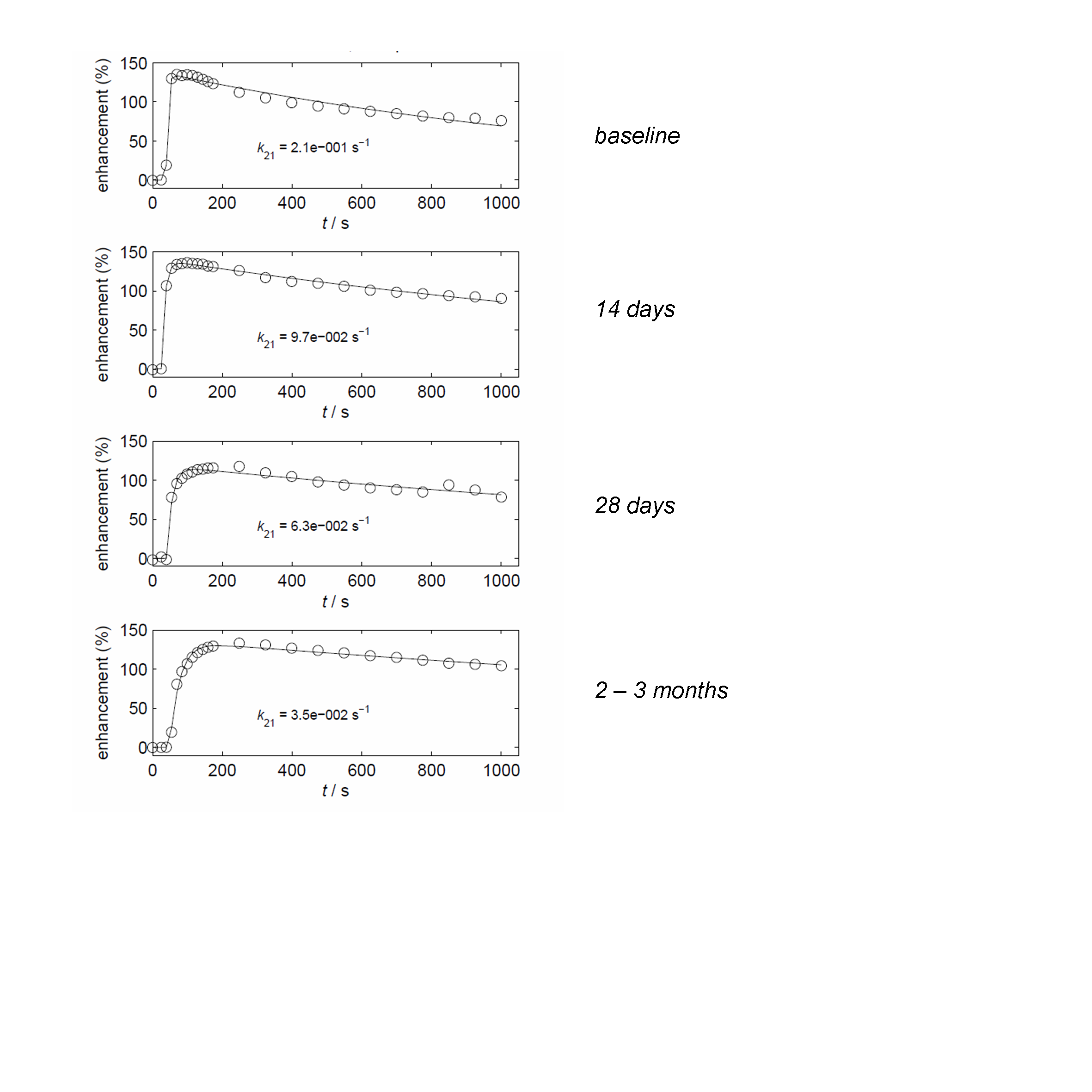

Supplement: Figure S1 — Example DCE-MRI data showing signal enhancement vs. time with fitting to the kinetic model described in the Materials and Methods section. Data from four serial scans from the same treated patient are displayed, showing reduction in the perfusion and permeability parameter k ep. (TIF) [file pone.0089809.s001.tif]

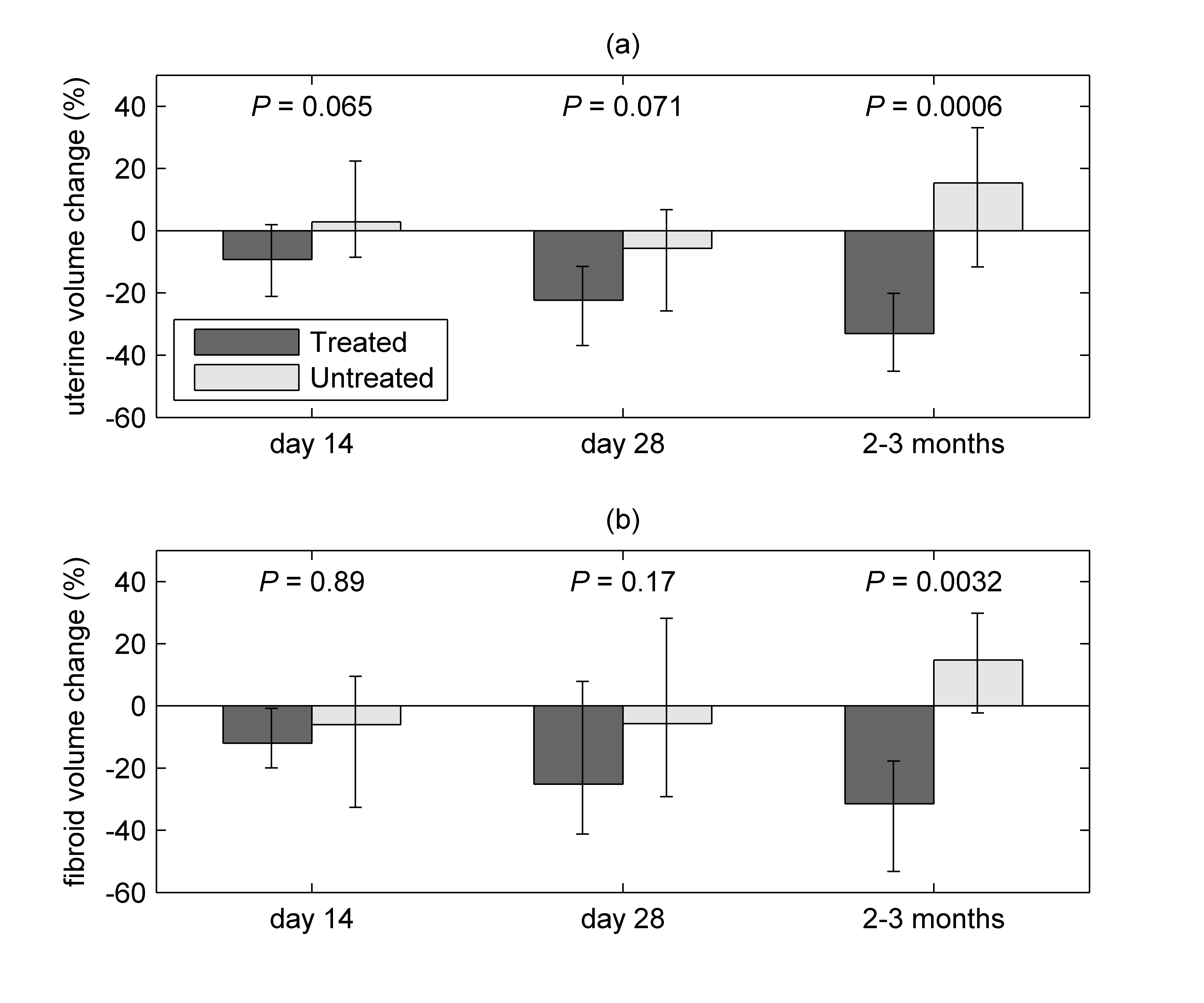

Supplement: Figure S2 — Median % volume change of (a) uterus and (b) largest fibroid from baseline at day 14, day 28 and 2 to 3 months (i.e. within 10 days of hysterectomy), measured by ultrasound; error bars show the interquartile range. Figure shows data for treated and untreated participants. (TIF) [file pone.0089809.s002.tif]

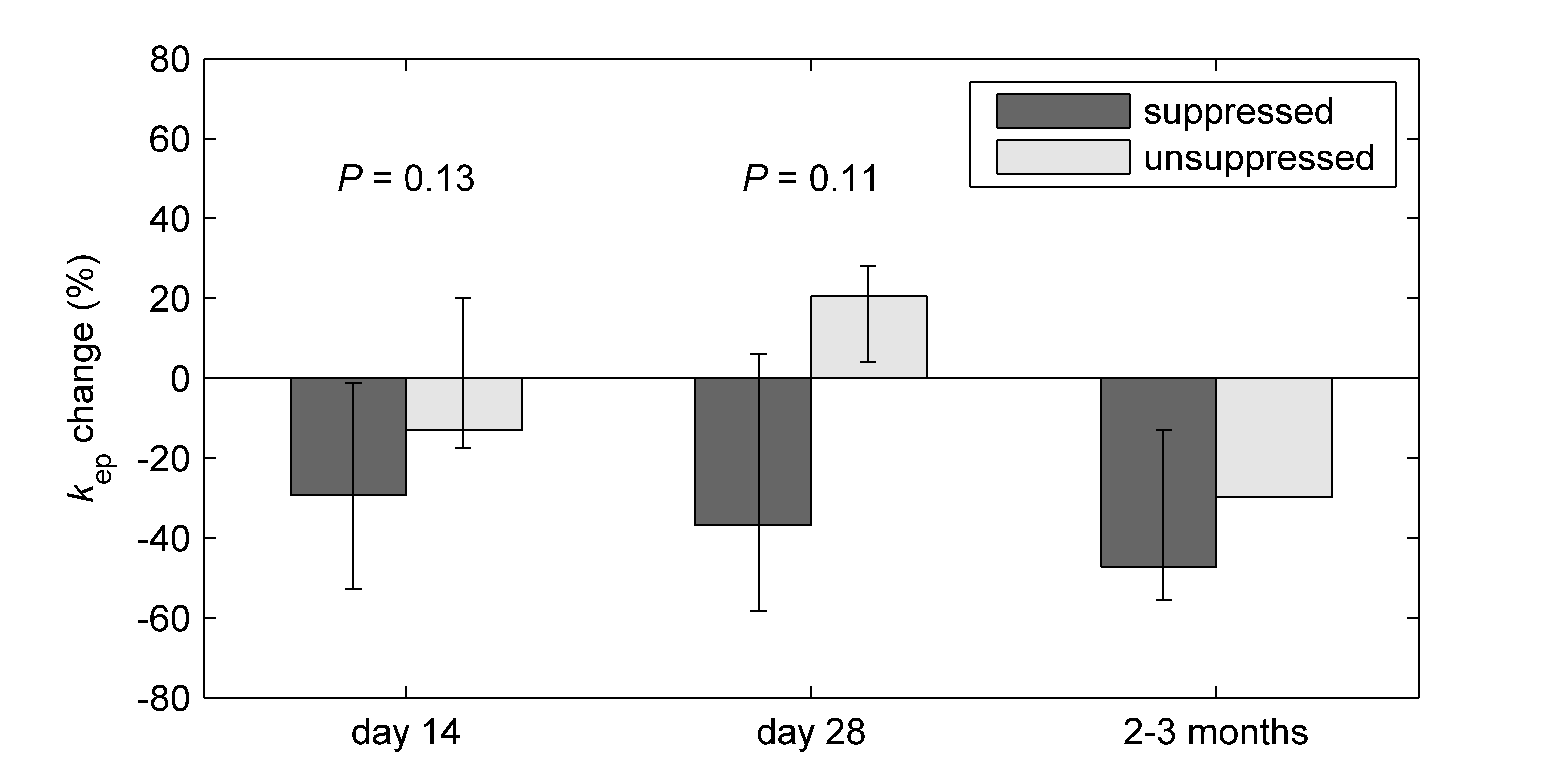

Supplement: Figure S3 — Median k ep change, assessed by DCE-MRI; error bars show the interquartile range. Figure shows data for treated participants, classified as oestradiol-suppressed and -unsuppressed as described in the text. (TIF) [file pone.0089809.s003.tif]
