# Supplementary material for: Quantitative Serial MRI of the Treated Fibroid Uterus
Source: PLoS One. 2014 Mar 7;9(3):e89809. doi: 10.1371/journal.pone.0089809 (PMC3946427; doi:10.1371/journal.pone.0089809)
Supplement: Table S1 — Adverse Events Log (*suspected unexpected serious adverse reaction (SUSAR); reported to MHRA as per protocol). (DOC) [file pone.0089809.s004.doc]

**Table S1:** Adverse Events Log (*suspected unexpected serious adverse reaction (SUSAR); reported to MHRA as per protocol)

| AE Description | AE Related (1=Yes, 2=No, 3=Possibly) | AE Severity (1=Mild, 2=Moderate, 3=Severe) |
| --- | --- | --- |
| Cold symptoms | 2 | 1 |
| Diarrhoea | 3 | 1 |
| Spots on face | 3 | 1 |
| Heavy vaginal bleeding | 3 | 1 |
| Headaches | 3 | 1 |
| Sore throat | 2 | 1 |
| Palpitations - 1 episode | 3 | 1 |
| Heavy vaginal bleeding with nausea and sweating | 3 | 1 |
| Insect bites | 2 | 1 |
| Raised CA125 blood level | 2 | 1 |
| Headaches | 3 | 1 |
| Sore throat | 2 | 1 |
| ***Heavy vaginal bleeding, drop in haemoglobin level, admitted to hospital overnight, required oral iron therapy** | **3** | **3** |
| Cold symptoms | 2 | 1 |
| Tooth infection | 2 | 1 |
| Reduced haemoglobin | 2 | 1 |
| Cough and sore throat | 2 | 1 |
| Headaches | 3 | 1 |
| Swine flu | 2 | 2 |
| Nose bleeds | 2 | 1 |
| Eye infection post laser eye surgery | 2 | 1 |
| Transient chest tightening in MRI scanner after administration of gadolinium. | 1 | 1 |
| Headache | 3 | 1 |
| 1 episode of palpitations and slight wheeze; resolved spontaneously. | 3 | 1 |
| Earache | 2 | 1 |
| Skin changes (facial spots) | 3 | 1 |
| 1 episode of palpitation with no associated chest pain or shortness of breath. | 3 | 1 |
| Left black eye | 2 | 1 |
| Stomach bug, nausea, diarrhoea | 2 | 1 |
| Cold symptoms | 2 | 1 |
| Raised blood pressure | 2 | 1 |
| Feeling transiently light-headed | 2 | 1 |
| Stiff neck | 2 | 1 |
| Flu-like symptoms, Headache, sore throat, muscle aches | 2 | 2 |
| Cough | 2 | 1 |
| Left eye infection | 2 | 1 |
| Cold symptoms | 2 | 1 |
| Muscle aches | 2 | 1 |
| Ear infection | 2 | 1 |
| Raised blood pressure | 2 | 1 |
| Candidiasis | 2 | 1 |
| Dry throat | 2 | 1 |
| Headache | 2 | 1 |
| Allergic reaction to gadolinium contrast | 1 | 2 |
| Cold symptoms | 2 | 1 |
| Abdominal pain | 2 | 2 |
| Heavy vaginal bleeding and pelvic pain | 2 | 2 |
| Elevated ALT level noted | 2 | 1 |
| Sty left eye | 2 | 1 |
| Abdominal pain | 2 | 1 |
| Pain in left calf | 2 | 1 |
| Fatigue | 2 | 1 |
