# Supplementary material for: Quantitative Serial MRI of the Treated Fibroid Uterus
Source: PLoS One. 2014 Mar 7;9(3):e89809. doi: 10.1371/journal.pone.0089809 (PMC3946427; doi:10.1371/journal.pone.0089809)
Supplement: Table S2 — Absolute median MTR change (%) from baseline. (DOC) [file pone.0089809.s005.doc]

**Table S2:** Absolute median MTR change (%) from baseline.

|  | day 14 | day 28 | 2-3 months |
| --- | --- | --- | --- |
| treated (%) | 0.4 | 0.3 | 0.4 |
| untreated (%) | -0.8 | -0.5 | -0.1 |
| *P* | 0.22 | 0.58 | 0.65 |
