# Supplementary material for: Quantitative Serial MRI of the Treated Fibroid Uterus
Source: PLoS One. 2014 Mar 7;9(3):e89809. doi: 10.1371/journal.pone.0089809 (PMC3946427; doi:10.1371/journal.pone.0089809)
Supplement: Text S1 — Description of Adverse Events and Missing Data. (DOC) [file pone.0089809.s006.doc]

***Adverse Events (see also Table S1)***

One patient (Group 1) experienced a suspected unexpected serious adverse reaction (SUSAR) following the second dose of GnRH agonist. The subject experienced an episode of excessively heavy vaginal bleeding resulting in anaemia and overnight hospital admission. The patient remained in the study to the endpoint of hysterectomy but received oral iron supplementation and the third dose of the GnRH agonist was omitted. The most common adverse events were coryzal symptoms, skin changes and headaches that were considered unrelated to study intervention. One patient (Group 1) had a raised Ca-125 tumour marker and due to an ovarian endometrioma that was removed at the time of hysterectomy. Three participants (one from each group) had anaemia at screening and were treated with oral iron supplementation.

***Missing data***

DCE-MRI data were unavailable for three patients within the untreated group: one patient had no venous access; in another patient the intravenous cannula fell out at visit 1 midway through infusion of contrast, rendering perfusion data incomplete; and a third patient had an adverse reaction to the injection of contrast, with symptoms of dizziness and breathlessness, which resolved spontaneously and did not require hospital admission. MT-MRI data were unavailable at pre-treatment imaging for one patient due to incorrect implementation of the protocol. It was not possible to obtain uterine artery Doppler ultrasound measurements for two patients at one of their visits. In one patient (untreated group) venepuncture was impossible and no blood samples were collected; for two further patients there were one and two missing oestradiol measurements respectively.
