# Supplementary material for: Quantitative Serial MRI of the Treated Fibroid Uterus
Source: PLoS One. 2014 Mar 7;9(3):e89809. doi: 10.1371/journal.pone.0089809 (PMC3946427; doi:10.1371/journal.pone.0089809)
Supplement: Protocol S1 — Trial protocol. (DOC) [file pone.0089809.s007.doc]

**Study Protocol**

Novel Magnetic Resonance Imaging strategies as a non-invasive biomarker of vascular and extracellular matrix morphology in women with uterine fibroids

| Sponsor | Co-sponsored by University of Edinburgh and NHS Lothian |
| --- | --- |
| Funder | TMRC |
| Funding Reference Number | WHMSB-EU-131 |
| Chief Investigator | Professor Hilary O D Critchley |
| EudraCT Number | 2008-001731-36 |
| CTA Number | 01384/0210/001-0001 |
| MREC Number | 08/MRE00/30 |
| Clinicaltrials.gov identifier | NCT00746031 |
| Version Number and Date | Version 2 13th October 2009 |

contents

contents [2](#__RefHeading___Toc197412973)

protocol approval [4](#__RefHeading___Toc197412974)

list of abbreviations [5](#__RefHeading___Toc197412975)

summary [6](#__RefHeading___Toc197412976)

**1.** **INTRODUCTION** [7](#__RefHeading___Toc197412977)

**1.1** **BACKGROUND** [7](#__RefHeading___Toc197412978)

**1.2** **RATIONALE FOR STUDY** [8](#__RefHeading___Toc197412979)

**2.** **STUDY OBJECTIVES** [9](#__RefHeading___Toc197412980)

**2.1** **OBJECTIVES** [9](#__RefHeading___Toc197412981)

**2.1.1** **Primary Objective** [9](#__RefHeading___Toc197412982)

**2.1.2** **Secondary Objectives** [9](#__RefHeading___Toc197412983)

**2.2** **ENDPOINTS** [9](#__RefHeading___Toc197412984)

**2.2.1** **Primary Endpoint** [9](#__RefHeading___Toc197412985)

**2.2.2** **Secondary Endpoints** [9](#__RefHeading___Toc197412986)

**3.** **STUDY DESIGN** [9](#__RefHeading___Toc197412987)

**3.1** **OVERALL STUDY DESIGN AND PLAN** [9](#__RefHeading___Toc197412988)

**3.1.2 Measurements**  11

**3.2** **STUDY DURATION** [12](#__RefHeading___Toc197412989)

**3.3** **CRITERIA FOR STUDY DISCONTINUATION** [12](#__RefHeading___Toc197412990)

**3.4** **DEFINITION OF END OF STUDY** [13](#__RefHeading___Toc197412991)

**4.** **STUDY POPULATION** [13](#__RefHeading___Toc197412992)

**4.1** **NUMBER OF PARTICIPANTS** [13](#__RefHeading___Toc197412993)

**4.2** **INCLUSION CRITERIA** [13](#__RefHeading___Toc197412994)

**4.3** **EXCLUSION CRITERIA** [13](#__RefHeading___Toc197412999)

**5.** **PARTICIPANT SELECTION AND ENROLMENT** [13](#__RefHeading___Toc197413001)

**5.1** **IDENTIFYING PARTICIPANTS** [13](#__RefHeading___Toc197413002)

**5.1.1** **Identification of Subjects** [14](#__RefHeading___Toc197413003)

**5.2** **CONSENTING PARTICIPANTS** [14](#__RefHeading___Toc197413004)

**5.3** **SCREENING FOR ELIGIBILITY** [14](#__RefHeading___Toc197413005)

**5.4** **INELIGIBLE AND NON-RECRUITED PARTICIPANTS** [14](#__RefHeading___Toc197413006)

**5.5** **RANDOMISATION** [14](#__RefHeading___Toc197413007)

**5.5.1** **Randomisation** [14](#__RefHeading___Toc197413008)

**5.5.2** **Treatment Allocation** [14](#__RefHeading___Toc197413009)

**5.5.3** **Emergency Unblinding Procedures** [14](#__RefHeading___Toc197413010)

**5.5.4** **Premature Withdrawal** [14](#__RefHeading___Toc197413011)

**6.** **INVESTIGATIONAL MEDICINAL PRODUCT AND PLACEBO** [15](#__RefHeading___Toc197413012)

**6.1** **STUDY DRUG** [15](#__RefHeading___Toc197413013)

**6.1.1** **Study Drug Identification** [15](#__RefHeading___Toc197413014)

**6.1.2** **Study Drug Manufacturer** 15

**6.1.3** **Marketing Authorisation Holder** [15](#__RefHeading___Toc197413020)

**6.1.4** **Labelling and Packaging** [15](#__RefHeading___Toc197413021)

**6.1.5** **Storage** [15](#__RefHeading___Toc197413022)

**6.1.6** **Summary of Product Characteristics** [15](#__RefHeading___Toc197413023)

**6.2** **PLACEBO** [15](#__RefHeading___Toc197413024)

**6.3** **DOSING REGIME** [15](#__RefHeading___Toc197413025)

**6.4** **DOSE CHANGES** [16](#__RefHeading___Toc197413026)

**6.5** **PARTICIPANT COMPLIANCE** [16](#__RefHeading___Toc197413027)

**6.6** **OVERDOSE** [16](#__RefHeading___Toc197413028)

**6.7** **OTHER MEDICATIONS** [16](#__RefHeading___Toc197413029)

**6.7.1** **Permitted Medications** [16](#__RefHeading___Toc197413030)

**6.7.2** **Prohibited Medications** [16](#__RefHeading___Toc197413031)

**7.** **STUDY ASSESSMENTS** [16](#__RefHeading___Toc197413032)

**7.1** **SAFETY ASSESSMENTS** [16](#__RefHeading___Toc197413033)

**7.2** **STUDY ASSESSMENTS** [16](#__RefHeading___Toc197413034)

**7.2.1 Sonographic assessment** [16](#__RefHeading___Toc197413035)

**7.2.2 Colour Doppler imaging** [17](#__RefHeading___Toc197413037)

**7.2.3 Magnetic resonance (MR) imaging** [17](#__RefHeading___Toc197413038)

**7.2.4 Evaluation of uterine MR and uterine histology** [18](#__RefHeading___Toc197413039)

**7.2.5 Blood Sampling** 19

**7.2.6 Urine Collection** 19

**7.2.7 Menstrual Recording** 19

**8.** **DATA COLLECTION** [19](#__RefHeading___Toc197413040)

**9.** **STATISTICS AND DATA ANALYSIS** [19](#__RefHeading___Toc197413041)

**9.1** **SAMPLE SIZE CALCULATION** [19](#__RefHeading___Toc197413042)

**9.2** **PROPOSED ANALYSES** [19](#__RefHeading___Toc197413043)

**10.** **ADVERSE EVENTS** [20](#__RefHeading___Toc197413044)

**10.1** **DEFINITIONS** [20](#__RefHeading___Toc197413045)

**10.2** **DETECTING AEs AND SAEs** [21](#__RefHeading___Toc197413046)

**10.3** **RECORDING AEs AND SAEs** [21](#__RefHeading___Toc197413047)

**10.4** **EVALUATION OF AEs AND SAEs** [21](#__RefHeading___Toc197413048)

**10.4.1** **Assessment of Seriousness** [21](#__RefHeading___Toc197413049)

**10.4.2** **Assessment of Causality** [21](#__RefHeading___Toc197413050)

**10.4.3** **Assessment of Severity** [22](#__RefHeading___Toc197413051)

**10.4.4** **Assessment of Expectedness** [22](#__RefHeading___Toc197413052)

**10.5** **REPORTING OF SAEs/SARs/SUSARs** [22](#__RefHeading___Toc197413053)

**10.6** **REGULATORY REPORTING REQUIREMENTS** [22](#__RefHeading___Toc197413054)

**10.7** **FOLLOW UP PROCEDURES** [23](#__RefHeading___Toc197413055)

**11.** **PREGNANCY** [23](#__RefHeading___Toc197413056)

**12.** **TRIAL MANAGEMENT AND OVERSIGHT ARRANGEMENTS** [23](#__RefHeading___Toc197413057)

**12.1** **PROJECT MANAGEMENT GROUP** [23](#__RefHeading___Toc197413058)

**12.2** **TRIAL MANAGEMENT** [23](#__RefHeading___Toc197413059)

**12.3** **CENTRAL TRIAL OFFICE** [23](#__RefHeading___Toc197413060)

**12.4** **TRIAL STEERING COMMITTEE** [23](#__RefHeading___Toc197413061)

**12.5** **DATA MONITORING COMMITTEE** [23](#__RefHeading___Toc197413062)

**12.6** **INSPECTION OF RECORDS** [23](#__RefHeading___Toc197413063)

**12.7** **STUDY MONITORING** [24](#__RefHeading___Toc197413064)

**12.8** **RISK ASSESSMENT** [24](#__RefHeading___Toc197413065)

**12.8.1** **Potential Risks** [24](#__RefHeading___Toc197413066)

**12.8.2** **Minimising Risk** [24](#__RefHeading___Toc197413068)

**13.** **GOOD CLINICAL PRACTICE MODULE** [24](#__RefHeading___Toc197413069)

**13.1** **ETHICAL CONDUCT OF THE STUDY** [24](#__RefHeading___Toc197413070)

**13.2** **REGULATORY COMPLIANCE THE STUDY** [24](#__RefHeading___Toc197413071)

**13.3** **INVESTIGATOR RESPONSIBILITIES** [25](#__RefHeading___Toc197413072)

**13.3.1** **Informed Consent** [25](#__RefHeading___Toc197413073)

**13.3.2** **Study Site Staff** [25](#__RefHeading___Toc197413074)

**13.3.3** **Data Recording** [25](#__RefHeading___Toc197413075)

**13.3.4** **Investigator Documentation** [25](#__RefHeading___Toc197413076)

**13.3.5** **GCP Training** [25](#__RefHeading___Toc197413077)

**13.3.6** **Confidentiality** [25](#__RefHeading___Toc197413078)

**13.3.7** **Data Protection** [26](#__RefHeading___Toc197413079)

**14.** **STUDY CONDUCT RESPONSIBILITIES** [26](#__RefHeading___Toc197413080)

**14.1** **PROTOCOL AMENDMENTS** [26](#__RefHeading___Toc197413081)

**14.2** **PROTOCOL VIOLATIONS AND DEVIATIONS** [26](#__RefHeading___Toc197413082)

**14.3** **STUDY RECORD RETENTION** [26](#__RefHeading___Toc197413083)

**14.4** **END OF STUDY** [26](#__RefHeading___Toc197413084)

**14.5** **CONTINUATION OF DRUG FOLLOWING THE END OF STUDY** [26](#__RefHeading___Toc197413085)

**15.** **REPORTING, PUBLICATIONS AND NOTIFICATION OF RESULTS** [26](#__RefHeading___Toc197413086)

**15.1** **AUTHORSHIP POLICY** [26](#__RefHeading___Toc197413087)

**15.2** **PUBLICATION** [27](#__RefHeading___Toc197413088)

**15.3** **PEER REVIEW** [27](#__RefHeading___Toc197413089)

**16.** **REFERENCES** [27](#__RefHeading___Toc197413090)

APPENDIX 1: Summary of Product Characteristics [28](#__RefHeading___Toc197413091)

APPENDIX 2: Monitoring Plan [39](#__RefHeading___Toc197413093)

APPENDIX 3: Risk Assessment [40](#__RefHeading___Toc197413094)

APPENDIX 4: Study plan [41](#__RefHeading___Toc197413095)

protocol approval

**Novel Magnetic Resonance Imaging strategies as a non-invasive biomarker of** **vascular and extracellular matrix morphology in women with uterine fibroids**

EudraCT number 2008-001731-36

**Signatures**

|  |  |  |  |
| --- | --- | --- | --- |
| Chief Investigator | Signature |  | Date |
|  |  |  |  |
|  |  |  |  |
|  |  |  |  |
|  |  |  |  |
| Trial Statistician | Signature |  | Date |
|  |  |  |  |
|  |  |  |  |
|  |  |  |  |
|  |  |  |  |
|  |  |  |  |
|  |  |  |  |
|  |  |  |  |
|  |  |  |  |
|  |  |  |  |

list of abbreviations

| AE | Adverse event |
| --- | --- |
| BMI | Body Mass Index |
| CRF | Case report form |
| CTCAE | Common Terminology Criteria for Adverse Events |
| E | Estradiol |
| EC | Ethics Committee |
| ECTU | Edinburgh Clinical Trials Unit |
| ECM | Extracellular Matrix |
| eGFR | Estimated Glomerular Filtration Rate |
| FSH | Follicle stimulating hormone |
| GnRH | Gonadotropin releasing hormone |
| GP | General Practitioner |
| HMB | Heavy menstrual bleeding |
| ICH GCP | International Conference on Harmonization of Good Clinical Practice |
| LH | Luteinizing hormone |
| MRI | Magnetic Resonance Imaging |
| MTI | Magnetization transfer imaging |
| MTR | Magnetization transfer ratio |
| P | Progesterone |
| SAE | Serious adverse event |
| SOP | Standard Operating Procedure |
| TAS | Transabdominal Scan |
| TMRC | Translational Medicine Research Collaboration |
| TMRI | Translational Medicine Research Institute |
| TVU | Transvaginal ultrasound |
| UAE | Uterine artery embolisation |
| ULN | Upper Limit of Normal |
| USS | Ultrasound Scan |

summary

Uterine fibroids (leiomyomas) are present in up to 80% of women of reproductive age. Associated heavy menstrual bleeding is frequently an indication for surgery. The only medical therapies for treating uterine fibroids are GnRH agonists. Non-invasive biomarkers to monitor clinical response of these agents on reduction of fibroid size and reduction in bleeding are needed. This proposal will evaluate the biomarker potential of contrast-enhanced magnetic resonance (MR) imaging in women with fibroids treated with standard therapies that reduce menstrual bleeding and fibroid/uterine size prior to hysterectomy.

# **INTRODUCTION**

## **1.1 BACKGROUND**

Complaints of excessive blood loss impose a detrimental impact on quality of life for healthy women^1^. Annually in the UK 3.5 million workdays are lost^2^ and treatment costs for menstrual complaints exceed £65m. Heavy menstrual bleeding (HMB; menorrhagia) is by far the most common complaint in women with symptomatic uterine fibroids (also termed leiomyomas), and represents the major indication for surgical intervention (hysterectomy).

Fibroids may be present in as many as 80% of women of reproductive age. One in 4 Caucasian women report clinically significant symptoms attributable to the presence of fibroids. Prevalence peaks in the perimenopausal years. Obesity and an early menarche are known risk factors, likely due to the increased lifetime exposure to oestrogen^3^. The mechanism of HMB in women with fibroids is not understood.

To date there are no approved medical treatments for long-term management of women with symptomatic fibroids.

Current treatments for fibroids are surgical or invasive in nature. Surgical removal (myomectomy) and hysterectomy are mainstays of management. Uterine artery embolisation (UAE) was introduced ten years ago as an alternative to hysterectomy for the surgical management of fibroids. The efficacy of UAE as an alternative therapy to surgery for uterine fibroids has recently been demonstrated to require longer-term follow-up^4^.

GnRH-analogues are used for medical treatment of symptomatic uterine fibroids pre-surgery. After 3-6 months of treatment, the resultant hypo-oestrogenic state results in an average 20-50% decrease in uterine and fibroid size^5^. It is assumed that it is the hypoestrogenism that causes shrinkage of fibroids; however addition of tamoxifen actually increases fibroid growth^6^. Much of the shrinkage occurs in the first 2 weeks when the women are not hypoestrogenic. The immediate suppression of the pituitary achieved with GnRH antagonists, with no initial stimulatory effect is the main advantage over the GnRH agonists. GnRH antagonists have been developed with acceptable pharmacokinetic and safety profiles^7^ and use proposed for management of fibroids.

There is unmet need for a pharmacologic agent able to reduce excessive bleeding and other symptoms associated with increased uterine volume, which could prevent or significantly delay surgery without causing significant unwanted hypoestrogenic side effects and allow preservation of fertility.

This study involves the use of non-invasive MR imaging of uterine morphology and vasculature and will provide invaluable information as to whether such techniques could be used as an efficacy biomarker in clinical trials involving novel potential therapeutic agents for medical treatment of uterine fibroids. The development of such a biomarker will facilitate the assessment of drugs in development for uterine fibroid therapy and the study of uterine pathologies in general.

**Novel magnetic resonance imaging of uterine fibroids (leiomyomas) to inform on modulation of vasculature and fibroid morphology**

MR imaging is a useful and acceptable tool for assessing the presence, location and size of uterine leiomyomas *in vivo*^8,9,10^ The technology is non-invasive and does not involve ionizing radiation. Furthermore, MRI technologies are being developed to be able to resolve tissue detail to near cellular level^11^.

Improvement in MRI techniques, such as that provided by 3T imaging, could allow characterisation of change in uterine and fibroid volume, vasculature and structure after pharmacologic interventions (e.g. administration of GnRH-agonists^9^), with a better resolution compared to other imaging methods, such as Doppler US. Additional information may also be provided by using diffusion-weighted imaging (DWI)^12^ and magnetisation transfer imaging^11^.

Our goal is to test the validity of novel MR imaging modalities, such as dynamic contrast and magnetization transfer imaging as non-invasive biomarkers of volumetric, vascular, and structural dynamic changes in the disease progression after a medical intervention. We have previously used the technique of dynamic contrast imaging at a number of time points to measure perfusion and permeability parameters in brain tumours following dexamethasone treatment^13^. We have also used magnetization transfer imaging to look at structural changes occurring in periventricular white matter lesions in normal ageing^11^.

Examination of the macroscopic and microscopic features of hysterectomy specimens will allow correlation with the imaging results.

## **1.2 RATIONALE FOR STUDY**

The purpose of this study is to investigate and validate the potential use of structural, dynamic contrast and magnetization transfer MR imaging as a non-invasive biomarker for monitoring responses to medical interventions aimed at reduction of excessive menstrual bleeding and decrease in uterine/ fibroid volume.

Such MR imaging strategies should provide a method to visualize and quantify changes in:

1. uterus and fibroid volume;
2. uterine vascular density and perfusion;
3. vasculature and extracellular matrix content of fibroids -

in women with fibroids administered a gonadotrophin-releasing hormone agonist (GnRH-a), either alone or with initial administration of a GnRH antagonist, for adjunct therapy to reduce uterine bleeding prior to hysterectomy.

The proposed study design will also provide important mechanistic information on whether the reduction in blood flow of uterine/ fibroid vasculature and shrinkage of fibroids is dependent upon subjects being hypoestrogenic.

Comparisons of novel magnetic resonance imaging data of the fibroid uterus, pre- and post GnRHa, GnRH antagonist, with a comparative “control” group of women who receive no therapeutic intervention pre-hysterectomy, will be made.

We will employ (1) routine ultrasound and colour Doppler imaging techniques; (2) use MR imaging and explore a novel “pre-clinical” opportunity to examine ex-vivo 7T imaging and correlate imaging data with the histo/morphology of uterine specimens; and (3) undertake histo-morphology of the uterus removed at hysterectomy. We will evaluate whether imaging with MR is applicable for the study of modulation of morphology and cellular arrangements of tissue and vascular structures in the uterus, thereby informing mechanism of action of therapies.

Specifically, we will investigate the use of dynamic contrast imaging using standard contrast agents to characterize changes in fibroid perfusion and permeability after treatment. For example, perfusion parameters, such as time to peak, mean transit time etc. can be determined from the first pass of the bolus of contrast agent, while signal-time curves give information on tissue permeability. Finally, we shall also investigate the use of magnetization transfer imaging (MTI). This imaging modality provides an index, the magnetization transfer ratio (MTR), which measures the efficiency of the magnetization exchange between the relatively free water protons inside tissue and those bound to protein macromolecules in cellular membranes. MTR may therefore help differentiate fibrous from normal tissue and indicate pathological change in fibroid structure after treatment.

MTI has previously been used in brain imaging^13^. Its use in uterine imaging will be completely novel.

The evaluation of ex-vivo uteri has not been previously reported. This methodology could theoretically give almost cellular or histo-pathological level detail of pathology. This is relevant as spatial resolution increases with increasing field strength of MRI units, 3 Tesla (3T) will soon be the clinical standard. As MRI is non-ionising it can be used as a repeated study to assess early, efficacy of success of an intervention. The proposed ex vivo study will enhance the interpretation of correlations between 1.5/3T MR imaging data and the histo-morphology of the uterus/ fibroid(s). If longer term medical treatment strategies are to become a mainstay of clinical management of symptomatic fibroids it will be essential to have confidence in MR as a biomarker of response to treatment and resolution/ progression of disease. Thus far it has not been possible to determine when a fibroid exhibits concerning atypical features with either ultrasound or standard MR.

Importantly the proposal will permit investigation of the mechanism of action of the above therapeutic interventions. It is assumed that much of the decline in volume of fibroids following treatment with GnRH agonist is due to decline in oestrogen. In fact during the first 10-12 days there is an increase in oestrogen due to “flare”. In contrast following GnRH antagonist there is an immediate drop in oestrogen. This proposal provides an opportunity to investigate the role of hypoestrogenism as an important component of the therapeutic effect of these compounds. By taking serial imaging measurements pre-treatment, at 14+/- 3 days, 28+/- 3 days after starting treatment and up to 10 days before hysterectomy the dynamics of the changes in size, blood flow, perfusion and extracellular matrix can be studied.

# **STUDY OBJECTIVES**

## **2.1 OBJECTIVES**

### **2.1.1 Primary Objective**

##### To establish the feasibility and reproducibility of novel MR imaging techniques in the evaluation of treatment response in women with uterine leiomyomas (fibroids).

### **2.1.2 Secondary Objectives**

To provide mechanistic information on whether the reduction in blood flow of uterine/fibroid vasculature in shrinkage fibroids is dependent upon subjects being hypoestrogenic.

## **2.2 ENDPOINTS**

### **2.2.1 Primary Endpoint**

Feasibility and reproducibility of use of structural, dynamic contrast and magnetization transfer (MTI) MR imaging to visualize and quantify changes in:

1. Uterus and fibroid volume

### **2.2.2 Secondary Endpoints**

1. Uterine vascular perfusion

1. Vasculature and extracellular matrix (ECM content of fibroids)

3. Whether (a) reduction of blood flow of uterine and fibroid vasculature and (b) shrinkage of fibroid depends upon subject being hypoestrogenic.

NB. The study aims to establish feasibility and reproduce ability of novel MR imaging techniques in the evaluation of treatment response in women with uterine fibroids. Hence there is not an efficacy measurement required for any of the drug treatment options.

# **STUDY DESIGN**

## **3.1 OVERALL STUDY DESIGN AND PLAN**

**Also see Charts – Appendix 4**

**PILOT Groups:**

Prior to commencement of the Open Investigational study (detailed below) we will refine our novel MRI technical evaluations of volumetric measurements and perfusion imaging.

**Pilot Group A -**

We wish to study up to 15 women already scheduled for hysterectomy who are, as part of routine management, going to receive a GnRH agonist prior to their planned surgery. The hysterectomy will be performed 21-28 days from the last dose of GnRH agonist. The GnRH agonists will be given 28+/-3 days apart. .

After successful screening and Ultrasound

On TWO occasions, these being - prior to the patient receiving the GnRH agonist and pre-hysterectomy after 2 or 3 months on GnRH agonist therapy, we will perform:

(i) Novel 1.5T MR imaging (including structural, dynamic contrast and magnetization transfer imaging)

On up to TWO occasions, these being - prior to the patient receiving the GnRH agonist and pre-hysterectomy after 2 or 3 months on GnRH agonist therapy, we will perform:

(ii) Doppler Ultrasound scanning. If Visit 1 falls within 28days of screening then the screening scan will remain valid. If this is anticipated then Dopplers will be carried out at screening.

Once the uterus is removed at hysterectomy the uterus will

(iii) also be studied in a novel way in the higher resolution 7T MR scanner and observations correlated with the histo-morphological examination of the uterus.

Blood samples will be taken on 3 occasions - at Screening and Day 28 to assess Urea and Electrolytes (U&E's), Full Blood Count (FBC), and Liver Function Tests (LFT's) and on the day of Hysterectomy (Visit 3) to assess, as before, plus Oestradiol (E), Progesterone(P), Luteinising Hormone (LH) and Follicle Stimulating Hormone (FSH) These blood samples will be taken on scheduled study visits to the hospital.

**Pilot Group B -**

We will recruit up to 15 women already scheduled for hysterectomy who will not be receiving a GnRH agonist prior to planned surgery as part of their routine clinical management.

After successful screening and Ultrasound -

On ONE occasion, within 10 days of their scheduled hysterectomy we will perform:

(i) Novel 1.5T MR imaging (including structural, dynamic contrast and magnetization transfer imaging.

(ii) Doppler Ultrasound scanning. If Visit 1 falls within 28days of screening then the screening scan will remain valid. If this is anticipated then Dopplers will be carried out at screening.

Once the uterus is removed at hysterectomy the uterus will

(iii) also be studied in a novel way in the higher resolution 7T MR scanner and observations correlated with the histo-morphological examination of the uterus.

Blood samples will be taken on 2 occasions - at Screening to assess Urea and Electrolytes (U&E's), Full Blood Count (FBC), and Liver Function Tests (LFT's) and on the day of Hysterectomy (Visit 2) to assess as before plus Oestradiol (E), Progesterone(P), Luteinising Hormone (LH) and Follicle Stimulating Hormone (FSH). These blood samples will be taken on scheduled study visits to the hospital.

-Pilot Group C

We will recruit up to 5 women already scheduled for hysterectomy who will not be receiving a GnRH agonist prior to planned surgery as part of their routine clinical management.

After successful screening and Ultrasound -

On ONE occasion, within 10 days of their scheduled hysterectomy we will perform:

1. Novel 1.5T MR imaging (including structural, dynamic contrast and magnetization transfer imaging) on each patient
2. Novel 3T MR imaging on each patient
3. Doppler Ultrasound scanning. If Visit 1 falls within 28days of screening then the screening scan will remain valid. If this is anticipated then Dopplers will be carried out at screening.

Once the uterus is removed at hysterectomy the uterus will

(iv) also be studied in a novel way in the higher resolution 7T MR scanner and observations correlated with the histo-morphological examination of the uterus.

Blood samples will be taken on 2 occasions - at Screening to assess Urea and Electrolytes (U&E's), Full Blood Count (FBC), and Liver Function Tests (LFT's) and on the day of Hysterectomy (Visit 2) to assess as before plus Oestradiol (E), Progesterone(P), Luteinising Hormone (LH) and Follicle Stimulating Hormone (FSH). These blood samples will be taken on scheduled study visits to the hospital.

**Main Study:**

This will be an open investigational study. This study will be conducted in up to 30 women with uterine fibroids scheduled for hysterectomy. Patients will be allocated to 3 treatment groups (randomised). The randomisation scheme will be drawn up by an independent statistician, and the allocated treatment codes will be put into sealed consecutively numbered opaque envelopes. When a patient is randomised into the study, she will receive the treatment indicated in the next available envelope.

For approximately 12 weeks (Groups 1 and 3) and approximately 13 weeks (Group 2) prior to surgery (hysterectomy) the participants will receive one of 3 possible treatment options:

Group 1 n=10: a monthly sub-cutaneous injection of a GnRH agonist [Goserlin 3.6mg] (total of 3 injections) commencing from day 1-5 of menstrual cycle

Group 2 n=10: a GnRH antagonist [sub-cutaneous injection of Cetrorelix 3mg] given on 3 occasions (Day 1, 4 & 7 +/- 1 day) commencing day 1 to 5 of the menstrual cycle with a GnRH agonist (Goserelin 3.6mgs) thereafter monthly for 3 months, commencing day 7+/- 1 day.

Group 3 n =10: A control group of women with fibroids (no medication). Day 1 of the study will be a specified day between day 1 to 5 of their menstrual cycle.

NB – Monthly is defined by 28 days +/-3 days.

The hysterectomy will be performed 21-28 days from the last dose of GnRH agonist. The GnRH agonists can be given 28+/- 3 days apart. The GnRH analogue, Goserelin 3.6mg subcutaneous, and the 2^nd^ and 3^rd^ doses of cetrorelix 3mg subcutaneous, can be administered in the patients’ own homes if more convenient. However, dose one of cetrorelix must be given in the hospital with the patient observed for 30minutes after the drug is administered.

**3.1.2 Measurements:**

Comparisons will be made between the following imaging modalities:

1. Sonographic assessment, colour Doppler imaging.
2. 1.5T MR imaging, including structural, dynamic contrast and magnetization transfer imaging.
3. Ex-vivo imaging with 7T MR scanner correlated with uterine histo-morphology.

For imaging exercises there will be one record per patient for each measurement at each time-point. The largest fibroid will be selected and total uterine volume measured.

It is not possible to have patients “blinded” to treatment option. It will be possible for the statistician, pathologists, radiologists, technicians and non clinical staff to be “blinded” to the treatment allocation, to the results of previous time-points, and to the results of other measures at the same time-point.

Following successful screening procedures all the participants will have a further 5 visits to the RIE.

On each of the visits 1 to 4 every participant will have a MRI, Ultrasound and Doppler. If Visit 1 falls within 28days of screening then the screening scan will remain valid. If this is anticipated then Dopplers will be carried out at screening.

Visit 1 will be carried out prior to randomisation (Within 28 days of Day 1)

Visit 2 at 14 days+/- 3 days following commencement of treatment

Visit 3 at 28 days+/- 3 days following commencement of treatment

Visit 4 Within 10 days prior to scheduled hysterectomy

Visit 5 Day of Hysterectomy and Ex-vivo imaging with 7T MR scanner of uterus

Blood samples will be taken on 3 occasions - at Screening, Day 28+/- 3 days (Visit 3)and on the day of Hysterectomy (Visit 5) to assess Urea and Electrolytes (U&E's), Full Blood Count (FBC), and Liver Function Tests (LFT's). These blood samples will be taken on scheduled study visits to the hospital.

A 10ml blood sample will be obtained on 11 occasions (Group 2) and 10 occasions (Groups 1 & 3) to obtain an estimate of Oestradiol (E), Progesterone(P), Luteinising Hormone (LH) and Follicle Stimulating Hormone (FSH). A small aliquot of blood will be stored in the freezer in reserve in case of any laboratory processing difficulties.

Some of these blood samples will be taken at the RIE when they coincide with Scheduled Study Visits. The participants will be given the option to have the rest of the blood samples taken in their own home if this is more convenient to them.

From Visit 1 all participants in the Main Study will be asked to collect 5mls of urine twice weekly and store frozen in their freezer at home. These will be collected on a regular basis by research staff.

In addition, a daily menstrual diary to record bleeding patterns throughout the study, commencing from Visit 1, until day of hysterectomy (Visit 5), will be completed by participants.

The study will be conducted on an inpatient basis on the day of hysterectomy and therefore, the majority of visits will be on an outpatient basis in the setting of the Edinburgh Royal Infirmary and other NHS Lothian Gynaecology Services or in the participant’s home if more convenient.

## **3.2 STUDY DURATION**

Up to 30 patients will be enrolled in the randomised study and up to 35 patients will be enrolled in the pilot study. For women recruited, the accrual period will be approximately 24 months. The study duration for each randomised patient includes a screening visit followed by 5 further visits over a total time period of up to 20 weeks. The study duration for each pilot patient includes a screening visit followed by up to 3 further visits over a total time period of up to 20 weeks.

## **3.3 CRITERIA FOR STUDY DISCONTINUATION**

The progress of this study will be assessed by The University of Edinburgh/NHS Lothian. The study may be discontinued for safety concerns.

## **3.4 DEFINITION OF END OF STUDY**

For the purposes of this study the “end of study” is defined as the day the last patient completes the last study visit.

# **STUDY POPULATION**

## **4.1 NUMBER OF PARTICIPANTS**

Up to 65 women.

## **4.2 INCLUSION CRITERIA**

Subjects must fulfil all of the following criteria:

### aged >18 years.

### Scheduled for hysterectomy with a fibroid uterus

### At least one intramural non-pedunculated, submucosal or subserous fibroid with a diameter of ≥ 2cm or multiple small fibroids with a uterine volume of ≥ 200cm^3^ confirmed by transvaginal or abdominal ultrasonography

### Normal cervical cytology within 3 years.

### Ability to understand and willingness to sign the informed consent form

## **4.3 EXCLUSION CRITERIA**

Subjects are to be excluded from the study if they meet any of the following criteria:

### Pregnant or lactating women

1. Contra-indication to magnetic resonance scanning
2. Unable to tolerate the supine position
3. Contra-indication to any of the medications to be utilized in study including gadolinium in contrast medium for MRI imaging
4. Diabetes and/or renal / hepatic impairment (eGFR<60mls/per min)
5. Known history of allergic predisposition

# **PARTICIPANT SELECTION AND ENROLMENT**

## **5.1 IDENTIFYING PARTICIPANTS**

Only subjects who meet all inclusion and no exclusion criteria can be considered for enrolment in this study. No study-specific procedures can be performed until the patient has signed an informed consent form that has been approved by the Ethics Committee. All patients who are considered for participation in the study should be recorded on a screening log that will be maintained at the site.

- - 1. Identification of Subjects

Patients will only be identified with the full knowledge and co-operation of their Clinical Care Team. Patients will be recruited from the Gynaecological Services of NHS Lothian Hospitals and include those women with uterine fibroids scheduled for hysterectomy.

Potential study recruits will also be identified from the Gynaecological Outpatients Services in NHS Lothian and from the waiting list for women scheduled for hysterectomy for fibroids at NHS Lothian Hospitals.

## **5.2 CONSENTING PARTICIPANTS**

Study participants will be consented by the Study Clinical Investigators and the Clinical Research Fellow. All study participants will receive a detailed patient information sheet with ample opportunity to ask questions about involvement in the study.

## **5.3 SCREENING FOR ELIGIBILITY**

At an initial screening visit the following will be conducted:

1. Medical history recording concomitant medications and menstrual history.
2. Routine physical examination.
3. Recording of vital signs.
4. Clearview hcG™ pregnancy test.
5. Multistix 8 SG™urinalysis.
6. Height and weight measurement with calculation of BMI from the standardised equation kg/m^2^.
7. The following blood tests: full blood count, urea and electrolytes (including eGFR), liver function tests.

It will be checked that there is no contra-indication to administration of GnRH agonist /antagonist or gadolinium (diabetes/ renal/ hepatic impairment) venepuncture/cannulation and MRI (inability to lie in supine position and claustrophobia) or buscopan, which is given routinely to all patients having a pelvic MRI to enhance image quality.

Each patient must be available throughout the study period.

## **5.4 INELIGIBLE AND NON-RECRUITED PARTICIPANTS**

Ineligible and non-recruited participants will return to routine care and will receive their hysterectomy as planned.

## **5.5 RANDOMISATION**

### **5.5.1 Randomisation**

A randomisation scheme will be drawn up by an independent statistician and allocated treatment codes will be put into sealed consecutively numbered opaque envelopes. When a patient is randomised she will receive the treatment indicated in the next available envelope.

### **5.5.2 Treatment Allocation**

Treatment allocation will be as per the randomisation instruction.

### **5.5.3 Emergency Unblinding Procedures**

Not applicable.

### **5.5.4 Premature Withdrawal**

Patients have the right to withdraw from the study at any time for any reason. The Investigator has the right to withdraw patients from the study according to his/her discretion. The reason(s) for withdrawal from the study must be recorded in the CRF.

Criteria for terminating participation in the study are listed below:

1. Patient withdrawal of consent
2. Investigator’s discretion that it is in the best interest of the patient to withdraw
3. Intercurrent illness: a condition, injury, or disease that renders continuing the study unsafe or regular follow-up impossible
4. General or specific changes in the patient's condition that renders the patient ineligible
5. Termination of the clinical study by the sponsor

# **INVESTIGATIONAL MEDICINAL PRODUCT AND PLACEBO**

## **6.1 STUDY DRUG**

### **6.1.1 Study Drug Identification**

## GnRH agonist – Zoladex (goserelin)

## GnRH antagonist/GnRHa – Cetrotide (cetrorelix)

### **6.1.2 Study Drug Manufacturer**

## GnRH agonist - Zoladex

This will be supplied via the pharmacy and the maufacturer is Astra Zeneca.

## GnRH antag/GnRHa - Cetrotide

This will be supplied by the pharmacy and the manufacturer is MerckSerono.

### **6.1.3 Marketing Authorisation Holder**

The MA holder of Zoladex is Astra Zeneca (MA number PL17901/0064).

The MA holder of Cetrotide is Serono Europe Ltd (MA number EU/1/99/100/003).

### **6.1.4 Labelling and Packaging**

This study is not blinded. Both Zoladex and Cetrotide will be labelled and packaged as per the manufacturer’s supplied drug.

### **6.1.5 Storage**

Zoladex - Do not store above 25°C.

Cetrotide - Do not store above 25 °C. Keep the vial in the outer carton in order to protect from light.

### **6.1.6 Summary of Product Characteristics**

The Summary of Product Characteristics (SoPC) for each of the drugs is given in Appendix 1.

## **6.2 PLACEBO**

Not applicable

## **6.3 DOSING REGIME**

Pilot Group A GnRH agonist (Zoladex 3.6 mg subcutaneously monthly)

Pilot Group B No medication

Group 1 GnRH agonist only (Zoladex 3.6 mg subcutaneously monthly)

Group 2 GnRH antagonist (Cetrorelix 3 mg) given on 3 occasions ,day 1, 4+/- 1 day & 7+/- 1 day, commencing day 1 to 5 of the menstrual cycle, followed with GnRH agonist (Zoladex 3.6mg) thereafter monthly for 3 months, commencing day 7+/- 1 day.

Group 3 Control group – No medication

NB: Monthly is defined by 28 days +/- 3 days.

## **6.4 DOSE CHANGES**

There will be no dose changes during the study.

## **6.5 PARTICIPANT COMPLIANCE**

The drugs are administered in the clinic or in the patient’s home (except dose 1 cetrorelix) and refusal of dose will be recorded in the CRF.

## **6.6 OVERDOSE**

Since the drugs are physician administered the risk of overdose is low. In the event of overdose the manufacturer will be consulted.

## **6.7 OTHER MEDICATIONS**

### **6.7.1 Permitted Medications**

All other medications deemed necessary or standard of care are permitted.

### **6.7.2 Prohibited Medications**

There are no prohibited medications.

# **STUDY ASSESSMENTS**

## **7.1 SAFETY ASSESSMENTS**

There are safety assessments for -

Pilot Group A at Screening, Day 28+/- 3 days and on day of hysterectomy.

Pilot Group B at Screening and on day of hysterectomy.

Pilot Group C at Screening and on day of hysterectomy.

Groups 1 to 3 at Screening, Day 28+/- 3 days and on day of hysterectomy.

## **7.2 STUDY ASSESSMENTS**

See attached summaries of study plan at end of protocol (Appendix 4)

## **Sonographic assessment**

All patients will be asked to participate in the following study imaging assessments

### Transvaginal ultrasound [TVU] (and abdominal ultrasound [TAS], if necessary) to assess fibroid size and location. The volumes of the largest leiomyoma and the uterus will be measured based on an estimation of the volume of an ellipsoid (V=πLDW/6 cm^3^) where L, W, and D represent length, width, and depth of the largest fibroid or the uterus.

### Subsequent scans (TVU / TAS) will be performed following start of medication at Visits 2, 3 and 4 (main study), Day 28+/- 3 days (Pilot Group A) and prior to hysterectomy (Pilot Groups A, B & C).and the same measurements will be carried out to record any changes from baseline.

## **7.2.2 Colour Doppler imaging**

To determine blood flow of the main uterine arteries colour Doppler imaging will be performed prior to the first study drug dose and repeated following start of medication at Visits 2, 3 and 4 (main study), Day 28+/- 3 days (Pilot Group A) and prior to hysterectomy (Pilot Groups A, B & C). Blood flow will be expressed using two impedance indices: the resistance index (RI) and the pulsatility index (PI). For each impedance index, the average of three measurements will be taken from the left and right arteries, respectively to enhance reproducibility.

## **7.2.3 Magnetic resonance (MR) imaging**

We aim to develop a novel non-invasive imaging protocol for serial examination of dynamic disease progression after therapeutic intervention (GnRH agonist, GnRH antagonist). Novel MR imaging strategies will include methods for characterisation of: uterine vascular density and perfusion; uterine fibroid volume; and assessment of cellular structure of fibroids.

Pilot Group A

Patients will be examined on 2 occasions:

##### (1) before commencing GnRH agonist (visit 1)

##### (2) just prior to hysterectomy(visit 2)

Pilot Group B

Patients will be examined on 1 occasion:

##### (1) just prior to hysterectomy(visit 2)

Pilot Group C

Patients will be examined on 1 occasion:

(1) just prior to hysterectomy (visit 2)

Main Study

##### Clinical MR imaging will be performed. Patients will be examined on four occasions:

##### (1) before commencing GnRH agonist, GnRH antagonist therapy (visit 1);

##### (2) after 14 days+/- 3 days (visit 2);

##### (3) after 28 days +/- 3 days (visit 3);

##### (4) within 10 days prior to hysterectomy(visit 4)

Pre- and post GnRH agonist / GnRH antagonist treatment and control subjects will undergo breath-hold fast-spin-echo T_2_-weighted imaging acquired in planes oblique to the uterus, magnetization transfer, contrast-enhanced T_1_-volume scans and dynamic contrast imaging using a phased array torso coil on MR scanner.

Dynamic contrast imaging will use T_1_-weighted SPGR scans acquired at a number of time points after injection of gadolinium, e.g. τ = 0, 30, 60, 90, 120 180, 240 and 300 seconds (Have practical limits in that patients usually become restless after about 30-40 minutes in MRI). Development work will be required to determine the optimum number and temporal resolution for this imaging to allow analysis of fibroid perfusion and permeability. Subjects will be carefully positioned so that the scan locations are as close as possible between pre- and post-treatment examinations. The use of image registration methods, such as FLIRT (www.fmrib.ox.ac.uk/fsl), to allow further fine alignment of imaging data will be investigated.

Uterus and enhancing and non-enhancing fibroid volumes pre- and post treatment will be determined from the T_2_- and T_1_-weighted structural scans using ANALYZE (Mayo Foundation, Rochester, MN, USA). Reduction in uterus and fibroid volumes will be determined as 100 × (Vol_pre_ – Vol_post_)/Vol_pre_.

Uterine and fibroid vascular density will be determined from signal-time curves generated from the dynamic contrast imaging data. Following the protocol described by Jha et al. (2000)^10^, regions-of-interest (ROI) will be placed over fibroids and a control region, for example myometrium, and signal-time curves produced. The ratio of fibroid to myometrrium signal intensity will then be calculated for each of the time points τ, and the maximum value determined. This maximum ratio value will then be used to determine the effect of treatment on fibroid vascularity from the relation 100 × (Ratio_pre_ – Ratio_post_)/Ratio_pre_. The application of image analysis protocols developed in the SFC Brain Imaging Centre, University of Edinburgh, to measure perfusion and permeability parameters from signal-time curves in brain tumours, will also be investigated in fibroids^13^.

Finally, we will investigate whether magnetisation transfer MRI (MTI) can provide a non-invasive biomarker to measure the clinical response of fibroids to treatment. This imaging modality provides an index, the magnetization transfer ratio (MTR), which measures the efficiency of the magnetization exchange between the relatively free water protons inside tissue and those bound to protein macromolecules in cellular membranes. We hypothesise that any pathological change in fibroid structure that involves alterations to cellular membrane macromolecules will change MTR and thus differentiate fibrous from normal tissue. We have previously used this technique to investigate the effects of ageing on the brain’s white matter^11^.

Development work will be required to determine the optimum imaging sequence parameters to provide MTI contrast in the uterus.

The examination of the uterus will include examination in the Preclinical Magnetic Resonance Imaging Unit (situated in the University of Edinburgh, Chancellors Building, at Little France site) using a 7T scanner. The inner diameter of the coil is 15cm thus able to accommodate the uterus once removed at hysterectomy. High resolution scans (T1W, T2W and MTI) will be carried out for comparison with histology.

## **7.2.4 Evaluation of uterine MR and uterine histology**

Pilot and Main Study

Both the endometrial and myometrial/leiomyoma compartments of the uterus will examined in response to medication.

Hysterectomy specimens will be examined by a sub-specialist gynaecological pathologist or a nominated deputy. After removal of the uterus in the operating theatre, an endometrial Pipelle biopsy will be taken in theatre and placed in RNA later. The uterus will then be transferred without delay to the pathology laboratory. The uterus will be ‘sliced’ in the plane in which it will be scanned. Biopsies will be taken of myometrium and leiomyoma and placed in RNA later. Formalin will then be instilled to fix the endometrium, the cervix will be plugged, the uterus will be securely packaged and escorted by member of research team to the MR imaging facility for ex vivo examination as described above. Thereafter the uterus will be escorted back to the laboratory for the whole specimen to be placed in formalin and fixed overnight. It will subsequently be sliced in the same plane as the MRI images, photographed and histology carried out.

**7.2.5 Blood Sampling**

Pilot Group A

Blood samples will be taken on 3 occasions -

At Screening, Day 28+/- 3 days (to assess Urea and Electrolytes (U&E's), Full Blood Count (FBC), and Liver Function Tests (LFT's). and on the day of Hysterectomy (Visit 3) to assess as per previous visits plus Oestradiol (E), Progesterone(P), Luteinising Hormone (LH) and Follicle Stimulating Hormone (FSH)

Pilot Group B

Blood samples will be taken on 2 occasions -

At Screening (to assess Urea and Electrolytes (U&E's), Full Blood Count (FBC), and Liver Function Tests (LFT's) and on the day of Hysterectomy (Visit 2) to assess as per previous visits plus Oestradiol (E), Progesterone(P), Luteinising Hormone (LH) and Follicle Stimulating Hormone (FSH)

Pilot Group C

Blood samples will be taken on 2 occasions -

At Screening (to assess Urea and Electrolytes (U&E's), Full Blood Count (FBC), and Liver Function Tests (LFT's) and on the day of Hysterectomy (Visit 2) to assess as per previous visits plus Oestradiol (E), Progesterone(P), Luteinising Hormone (LH) and Follicle Stimulating Hormone (FSH)

Main Study

A 10ml blood sample will be obtained on 11 occasions (Group 2) and 10 occasions (Groups 1 & 3) to obtain an estimate of Oestradiol (E), Progesterone(P), Luteinising Hormone (LH) and Follicle Stimulating Hormone (FSH). Urea and Electrolytes (U&E's), Full Blood Count (FBC), and Liver Function Tests (LFT's) will be checked at screening, Day 28+/- 3 days (Visit 3) and on the day of Hysterectomy (Visit 5).

- - 1. **Urine Collection**

Main Study only

5mls urine samples collected twice weekly to measure sex steroid hormone metabolites. Up to 30 samples in total. Instructions will be given to participants. Patient is asked at each point of contact if collection is being completed and if more sample bottles are required. A small drop if glycerol is put into the sample bottles to help with the freezing process. This has no effect on the analysis of the sample.

**7.2.7 Menstrual Recording**

Main Study Only

A daily record of menstrual bleeding maintained by the participant. This is given to patients at Visit 1 and collected on day of hysterectomy. Patients are asked at each point of contact if chart is being completed.

# **DATA COLLECTION**

CRFs have been created by research staff. Clinical research assistant, nurses or Clinical Research Fellow will collect data on CRFs. Data will be collected at each point of contact with participant.

# **STATISTICS AND DATA ANALYSIS**

## **9.1 SAMPLE SIZE CALCULATION**

No sample size required for pilot group.

Sample size calculations for this sort of study are very difficult. This is a hypothesis generating study, and findings will need to be confirmed in a separate larger study. A sample size of 10 patients in each group will give an 80% power, at a significance level of 5%, to detect a difference in the change in volume of 160cm cubed (s.d of change in volume = 120cm cubed) between a treated group and the control group. This calculation does not take multiple comparisons into account.

In terms of agreement 30 subjects would allow us to estimate limits of agreement +/- 0.6 standard deviations of the difference between measurements using Bland-Altman methods.

## **9.2 PROPOSED ANALYSES**

We will perform per protocol and Intention to Treat (ITT) analyses. Standard statistical analysis packages will be used. Given the limited sample size, only descriptive statistics will be used. This is however a hypothesis generating study and the statistical analyses performed will be within this context.

Missing data will be kept to a minimum, and where possible will be collected from patients regardless of whether they have been compliant with their allocated treatment.  Statistical analyses will involve sensitivity analyses to assess the effect of missing data.  We will collect reasons for missing data.

The randomisation scheme will be drawn up by an independent statistician, and the allocated treatment codes will be put into sealed consecutively numbered opaque envelopes.  When a patient is randomised into the study, she will receive the treatment indicated in the next available envelope.

**Primary analyses will be:**
(1) the comparison of the pre-hysterectomy uterine volume between the treated and control groups using t-test or a non-parametric equivalent as appropriate.:

(2) Bland-Altman analyses comparing the pre-hysterectomy MR with USS.

**Secondary analyses**: We will perform similar analyses for uterine vascular perfusion. A variety of other exploratory analyses will be performed including examining changes over time, and quality of images. Data will also be considered in the context of the patient’s hormone profile over the time of the study (taken from blood and urine samples).

# **ADVERSE EVENTS**

The Investigator is responsible for the detection and documentation of events meeting the criteria and definitions detailed below.

Full details of contraindications and side effects that have been reported following administration of the trial drug can be found in the relevant Summary of Product Characteristics (SoPC) in Appendix 1.

Participants should be instructed to contact their Investigator at any time after consenting to join the trial if any symptoms develop. All adverse events (AEs) that occur after joining the trial must be reported in detail in the CRF. In the case of an AE, the Investigator should initiate the appropriate treatment according to their medical judgment. Participants with AEs present at the last visit must be followed up until resolution of the event.

Any abnormal measurement/finding from any of our clinical interventions (eg patient observations, urine, blood, imaging) will be dealt with appropriately in a standard clinical way.

Any complications which arise at or after hysterectomy-excess blood loss, post operative pyrexia/infection, damage to bladder/bowel or deep vein thrombosis will not be recorded as adverse events within this study. These are recognised complications of hysterectomy which are discussed routinely between the surgeon and the patient when the operation consent form is completed.

## **10.1 DEFINITIONS**

An **adverse event** (AE) is any untoward medical occurrence in a clinical trial subject who is administered a medicinal product, which does not have a causal relationship with the treatment.

An **adverse reaction** (AR) is any untoward or unintended response to an investigational medicinal product related to any dose administered.

An **unexpected adverse reaction** (UAR) is an adverse reaction that is not consistent with the product information in the SoPC.

A **serious adverse event** (SAE), **serious adverse reaction** (SAR) or **suspected unexpected serious adverse reaction** (SUSAR) is any AE, AR or UAR that at any dose:

- results in death;
- is life threatening (i.e. the subject was at risk of death at the time of the event; it does not refer to an event which hypothetically might have caused death if it were more severe);
- requires hospitalisation or prolongation of existing hospitalisation;
- results in persistent or significant disability or incapacity;
- is a congenital anomaly or birth defect.

Note: Hospitalisations for treatment planned prior to randomisation and hospitalisation for elective treatment of a pre-existing condition will not be considered as an AE. Complications occurring during such hospitalisation will be AEs.

## **10.2 DETECTING AEs AND SAEs**

All AEs and SAEs must be recorded from the time a participant consents to join the study until the last study visit.

The Investigator should ask about the occurrence of AEs/SAEs at every visit during the study. Open-ended and non-leading verbal questioning of the participant should be used to enquire about AE/SAE occurrence. Participants should also be asked if they have been admitted to hospital, had any accidents, used any new medicines or changed concomitant medication regimens. If there is any doubt as to whether a clinical observation is an AE, the event should be recorded.

## **10.3 RECORDING AEs AND SAEs**

When an AE/SAE occurs, it is the responsibility of the Investigator to review all documentation (e.g. hospital notes, laboratory and diagnostic reports) related to the event. The Investigator should then record all relevant information in the CRF and on the SAE form (if the AE meets the criteria of serious).

Information to be collected includes dose, type of event, onset date, Investigator assessment of severity and causality, date of resolution as well as treatment required, investigations needed and outcome. MedDRA will be used to code all AEs.

## **10.4 EVALUATION OF AEs AND SAEs**

This is not a blinded trial therefore seriousness, causality, severity and expectedness should be evaluated on a patient to patient basis.

### **10.4.1 Assessment of Seriousness**

The Investigator should make an assessment of seriousness as defined in Section 10.1.

### **Assessment of Causality**

The Investigator must make an assessment of whether the AE/SAE is likely to be related to treatment according to the following definitions. All AEs/SAEs judged as having a reasonable suspected causal relationship (e.g. possibly, probably, definitely) to the study drug will be considered as ARs/SARs. If concomitant or rescue/escape drugs are given, the Investigator must also make an assessment of whether the AE/SAE is likely to be related to an interaction between the study drug and concomitant or rescue/escape drugs or whether the AE/SAE might be linked to either the study drug or concomitant or rescue/escape drugs but cannot be attributed to only one of these drugs. All AEs/SAEs judged as being related (e.g. possibly, probably, definitely) to an interaction between the study drug and concomitant or rescue/escape drugs, or any AE/SAE that cannot be attributed to only the study drug or the concomitant or rescue/escape drugs will also be considered to be ARs/SARs .

**Unrelated**: where an event is not considered to be related to the study drug.

**Possibly**: although a relationship to the study drug cannot be completely ruled out, the nature of the event, the underlying disease, concomitant medication or temporal relationship make other explanations possible.

**Probably**: the temporal relationship and absence of a more likely explanation suggest the event could be related to the study drug.

**Definitely**: The known effects of the study drug or its therapeutic class, or based on challenge testing, suggest that study drug is the most likely cause.

Alternative causes such as natural history of the underlying disease, concomitant therapy, other risk factors and the temporal relationship of the event to the treatment should be considered and investigated. The blind should not be broken for the purpose of making this assessment.

### **10.4.3 Assessment of Severity**

The Investigator should make an assessment of severity for each AE/SAE and record this on the CRF according to one of the following categories:

**Mild**: an event that is easily tolerated by the participant, causing minimal discomfort and not interfering with every day activities.

**Moderate**: an event that is sufficiently discomforting to interfere with normal everyday activities.

**Severe**: an event that prevents normal everyday activities.

Note: the term ‘severe’, used to describe the intensity, should not be confused with ‘serious’ which is a regulatory definition based on participant/event outcome or action criteria. For example, a headache may be severe but not serious, while a minor stroke is serious but may not be severe.

### **10.4.4 Assessment of Expectedness**

If an event is judged to be an AR/SAR, the evaluation of expectedness should be made based on knowledge of the reaction and the relevant product information documented in the SoPC.

## **10.5 REPORTING OF SAEs/SARs/SUSARs**

Once the Investigator becomes aware that an SAE has occurred in a study participant, they must report the information to the Edinburgh Clinical Trials Unit (ECTU) within 24 hours. The SAE form must be completed as thoroughly as possible with all available details of the event, signed by the Investigator or designee. If the Investigator does not have all information regarding an SAE, they should not wait for this additional information before notifying ECTU. The form can be updated when the additional information is received.

The SAE report must provide an assessment of causality and expectedness at the time of the initial report to ECTU according to Sections 10.4.2, Assessment of Causality and 10.4.4, Assessment of Expectedness.

The SAE form should be transmitted by fax to the ECTU central office on 0131 242 9447 or may be transmitted by hand to the office.

## **10.6 REGULATORY REPORTING REQUIREMENTS**

ECTU is responsible for Pharmacovigilance reporting on behalf of the Co-Sponsors (Edinburgh University and Lothian Health Board).

ECTU has a legal responsibility to notify the regulatory competent authority and the relevant ethics committee (main Research Ethics Committee (REC) that approved the trial). Fatal or life threatening SUSARs will be reported no later than 7 calendar days and all other SUSARs will be reported no later than 15 calendar days after ECTU is first aware of the reaction.

An Annual Safety Report will be submitted to the regulatory competent authority and the main REC listing all SARs and SUSARs.

## **10.7 FOLLOW UP PROCEDURES**

After initially recording an AE or recording and reporting an SAE, the Investigator is required to follow each participant until resolution. Follow up information on an SAE should be reported to ECTU.

AEs still present in participants at the last study visit should be monitored until resolution of the event or until no longer medically indicated.

# **PREGNANCY**

Pregnancy is not considered an AE or SAE, however, the Investigator must collect pregnancy information on any female participants or female partners of male participants who become pregnant while participating in the study. The Investigator should record the information on a Pregnancy Notification Form and submit this to ECTU within 14 days of being made aware of the pregnancy.

All pregnant female participants and partners of male participants should be followed up until following the birth or otherwise (i.e. spontaneous termination) to allow information on the status of the mother and child to be reported to ECTU.

# **TRIAL MANAGEMENT AND OVERSIGHT ARRANGEMENTS**

## **12.1 PROJECT MANAGEMENT GROUP**

The trial will be coordinated by a Project Management Group, consisting of the grant-holders (Chief Investigator and Co-Investigators), Clinical Research Fellow and Study Research Assistant/Nurse. Trial management will be the role of the Study Research Assistant/Nurse.

## **12.2 TRIAL MANAGEMENT**

Study Research Assistant/Nurse will oversee day to day running of the study and will be accountable to the Chief Investigator; be responsible for checking the CRFs for completeness, plausibility and consistency. Any queries will be resolved by the Investigator or delegated member of the trial team.

A Delegation Log will be prepared, detailing the responsibilities of each member of staff working on the trial.

## **12.3 CENTRAL TRIAL OFFICE**

The Central Trial Office is based in the Edinburgh Clinical Trials Unit (ECTU) and will provide support to the study.

## **12.4 TRIAL STEERING COMMITTEE**

A Trial Steering Committee (TSC) is not required.

This trial will be monitored by the Co-Sponsors for compliance to GCP, the Clinical Trial Regulations and the protocol.

## **12.5 DATA MONITORING COMMITTEE**

A Data Monitoring Committee (DMC) is not required.

The GnRH agonist Zoladex is prescribed within licence.

Cetrotide is licenced for use in assisted reproduction; use as described in this protocol is to suppress gonadotrophin release in a rapid manner.

SAEs/SUSARs will be reported to the Sponsor (this is monitored as part of the reporting system).

## **12.6 INSPECTION OF RECORDS**

Principal and co-Investigators and institutions involved in the study will permit trial related monitoring, audits, REC review, and regulatory inspection(s). In the event of an audit, the Investigator agrees to allow the Sponsor, representatives of the Sponsor or regulatory authorities direct access to all study records and source documentation.

## **12.7 STUDY MONITORING**

The ACCORD (joint office for University of Edinburgh and Lothian Health Board) Clinical Trials Monitor or an appointed local monitor will visit the Edinburgh study site prior to the start of the study and during the course of the study.

A copy of the monitoring plan is given in Appendix 2.

## **12.8 RISK ASSESSMENT**

An independent risk assessment carried out by the ACCORD Clinical Trials Monitor is given in Appendix 3.

### **12.8.1 Potential Risks**

## GnRH agonist – Zoladex (Goserelin)

Cetrotide

See Summary of Product Characteristics in Appendix 1.

Administration of these medications above should not present risks over and above normal clinical care.

Venepuncture and subcutaneous injections may cause very minimal discomfort and bruising.

Magnetic Resonance Imaging (MRI) can induce symptoms of claustrophobia in susceptible people.

Gadolinium- The standard dose which we will be using is 0.2mg/kg. In a very small percentage of cases people may experience hypersensitivity or an allergic reaction to gadolinium. Side effects, too are very rare but can include mild headache,

light headedness, urticaria, wheezing, nausea and vomiting and local pain. If eGFR is normal at screening then it is unlikely that there will be any change to renal function after two or three doses of the gadolinium contrast. Renal function will, however be reassessed at the time of the third MR scan.

Buscopan-The standard dose which we will be using is 20mg by slow intravenous injection. Side effects are uncommon but can include constipation, transient bradycardia, reduced bronchial secretions, urinary urgency and retention, dilatation of the pupils, dry mouth, flushing and dryness of the skin.

### **12.8.2 Minimising Risk**

Anxiety will be minimised by offering a full explanation of what to expect prior to scan and maintaining verbal contact during MR scan.

A thorough medical history will be taken at time of screening to assess for any contraindications to the study drugs.

# **GOOD CLINICAL PRACTICE MODULE**

## **13.1 ETHICAL CONDUCT OF THE STUDY**

The study will be conducted in accordance with the principles of the International Conference on Harmonisation Tripartite Guideline for Good Clinical Practice (ICH GCP).

A favourable ethical opinion will be obtained from the appropriate REC and local R&D approval will be obtained prior to commencement of the study.

## **13.2 REGULATORY COMPLIANCE THE STUDY**

The study will not commence until a Clinical Trial Authorisation (CTA) is obtained from the appropriate Regulatory Authority. The protocol and study conduct will comply with the Medicines for Human Use (Clinical Trials) Regulations 2004, and any relevant amendments.

## **13.3 INVESTIGATOR RESPONSIBILITIES**

The Investigator is responsible for the overall conduct of the study at the site and compliance with the protocol and any protocol amendments. In accordance with the principles of ICH GCP, the following areas listed in this section are also the responsibility of the Investigator. Responsibilities may be delegated to an appropriate member of study site staff. Delegated tasks must be documented on a Delegation Log and signed by all those named on the list.

### **13.3.1 Informed Consent**

The Investigator is responsible for ensuring informed consent is obtained before any protocol specific procedures are carried out. The decision of a participant to participate in clinical research is voluntary and should be based on a clear understanding of what is involved.

Participants must receive adequate oral and written information – appropriate Participant Information and Informed Consent Forms will be provided. The oral explanation to the participant should be performed by the Investigator or designated person, and must cover all the elements specified in the Participant Information Sheet/Informed Consent.

The participant must be given every opportunity to clarify any points they do not understand and, if necessary, ask for more information. The participant must be given sufficient time to consider the information provided. It should be emphasised that the participant may withdraw their consent to participate at any time without loss of benefits to which they otherwise would be entitled.

The participant should be informed and agree to their medical records being inspected by regulatory authorities but understand that their name will not be disclosed outside the hospital.

The Investigator or delegated member of the trial team and the participant should sign and date the Informed Consent Form(s) to confirm that consent has been obtained. The participant should receive a copy of this document and a copy filed in the Investigator Site File (ISF).

### **13.3.2 Study Site Staff**

The Investigator must be familiar with the IMP, protocol and the study requirements. It is the Investigator’s responsibility to ensure that all staff assisting with the study are adequately informed about the IMP, protocol and their trial related duties.

### **13.3.3 Data Recording**

The Investigator is responsible for the quality of the data recorded in the CRF.

### **13.3.4 Investigator Documentation**

Prior to beginning the study, each Investigator will be asked to provide particular essential documents to ECTU, including but not limited to:

- An original signed Investigator’s Declaration (as part of the Clinical Trial Agreement documents);
- Curriculum vitae (CV), signed and dated by the Investigator indicating that it is accurate and current.

ECTU will ensure all other documents required by ICH GCP are retained in a Trial Master File and that appropriate documentation is available in local ISFs.

### **13.3.5 GCP Training**

All study staff must hold evidence of appropriate GCP training or undergo GCP training. This should be updated every two years throughout the trial.

### **13.3.6 Confidentiality**

All laboratory specimens, evaluation forms, reports, and other records must be identified in a manner designed to maintain participant confidentiality. All records must be kept in a secure storage area with limited access. Clinical information will not be released without the written permission of the participant, except as necessary for monitoring and auditing by the Sponsor, its designee, Regulatory Authorities, or the REC. The Investigator and study site staff involved with this study may not disclose or use for any purpose other than performance of the study, any data, record, or other unpublished, confidential information disclosed to those individuals for the purpose of the study. Prior written agreement from the Sponsor or its designee must be obtained for the disclosure of any said confidential information to other parties.

### **13.3.7 Data Protection**

All Investigators and study site staff involved with this study must comply with the requirements of the Data Protection Act 1998 with regard to the collection, storage, processing and disclosure of personal information and will uphold the Act’s core principles. Access to collated participant data will be restricted to those clinicians treating the participants.

Computers used to collate the data will have limited access measures via user names and passwords.

Published results will not contain any personal data that could allow identification of individual participants.

# **STUDY CONDUCT RESPONSIBILITIES**

## **14.1 PROTOCOL AMENDMENTS**

Any changes in research activity, except those necessary to remove an apparent, immediate hazard to the participant, must be reviewed and approved by the Chief Investigator. Amendments to the protocol must be submitted in writing to the appropriate REC, Regulatory Authority and local R&D for approval prior to participants being enrolled into an amended protocol.

## **14.2 PROTOCOL VIOLATIONS AND DEVIATIONS**

The Investigator should not implement any deviation from the protocol without agreement from the Chief Investigator and appropriate REC, Regulatory Authority and R&D approval except where necessary to eliminate an immediate hazard to trial participants.

In the event that an Investigator needs to deviate from the protocol, the nature of and reasons for the deviation should be recorded in the CRF. If this necessitates a subsequent protocol amendment, this should be submitted to the REC, Regulatory Authority and local R&D for review and approval if appropriate.

## **14.3 STUDY RECORD RETENTION**

All study documentation will be kept for 15 years.

## **14.4 END OF STUDY**

The end of study is defined as the last participant’s last visit.

The Investigators and/or the trial steering committee have the right at any time to terminate the study for clinical or administrative reasons.

The end of the study will be reported to the REC and Regulatory Authority within 90 days, or 15 days if the study is terminated prematurely. The Investigators will inform participants and ensure that the appropriate follow up is arranged for all involved.

A summary report of the study will be provided to the REC and Regulatory Authority within 1 year of the end of the study.

## **14.5 CONTINUATION OF DRUG FOLLOWING THE END OF STUDY**

Drugs will not be provided following the end of study as after hysterectomy any treatment with Zoladex or Cetrorelix would not be required for further clinical indication.

# **REPORTING, PUBLICATIONS AND NOTIFICATION OF RESULTS**

## **15.1 AUTHORSHIP POLICY**

Ownership of the data arising from this study resides with the study team. On completion of the study, the study data will be analysed and tabulated, and a clinical study report will be prepared in accordance with ICH guidelines.

## **15.2 PUBLICATION**

The clinical study report will be used for publication and presentation at scientific meetings. Investigators have the right to publish orally or in writing the results of the study.

Summaries of results will also be made available to Investigators for dissemination within their clinics (where appropriate and according to their discretion).

## **15.3 PEER REVIEW**

This study has been reviewed and approved by the Scientific Board of the Translational Medicine Research Collaboration (TMRC), the study funder.

# **REFERENCES**

1. Sculpher M. (1998) A cost-utility analysis of abdominal hysterectomy versus transcervical resection for the surgical treatment of menorrhagia*. Int J Technol Assess Health Care* 14:302-19.
2. Weeks AD, Duffy SR, Walker JJ. (2000) A double-blind randomised trial of leuprorelin acetate prior to hysterectomy for dysfunctional uterine bleeding. *Brit J Obstet Gynaecol* 107:323-8.
3. Walker CL, Stewart EA. (2005) Uterine fibroids: The elephant in the room. *Science* 308: 1589-1592.
4. Edwards RD, Moss JG, Murray GD. (2000) Uterine-artery embolization versus surgery for symptomatic uterine fibroids. *N Engl J Med* 356:360 -70.
5. Lethaby A, Vollenhoven B, Sowter M. (2001) Pre-operative GnRH analogue therapy before hysterectomy or myomectomy for uterine fibroids. *Cochrane Database Syst Rev*: CD000547.
6. West CP, Lumsden MA, Lawson S, Williamson J, Baird DT. (1987) Shrinkage of uterine fibroids during therapy with goserelin (Zoladex): a luteinizing hormone-releasing hormone agonist administered as a monthly subcutaneous depot. *Fertil Steril* 48:45-51.
7. [Schultze-Mosgau A](http://www.ncbi.nlm.nih.gov/sites/entrez?Db=pubmed&Cmd=Search&Term="Schultze-Mosgau A"%5BAuthor%5D&itool=EntrezSystem2.PEntrez.Pubmed.Pubmed_ResultsPanel.Pubmed_RVAbstractPlus), [Griesinger G](http://www.ncbi.nlm.nih.gov/sites/entrez?Db=pubmed&Cmd=Search&Term="Griesinger G"%5BAuthor%5D&itool=EntrezSystem2.PEntrez.Pubmed.Pubmed_ResultsPanel.Pubmed_RVAbstractPlus), [Altgassen C](http://www.ncbi.nlm.nih.gov/sites/entrez?Db=pubmed&Cmd=Search&Term="Altgassen C"%5BAuthor%5D&itool=EntrezSystem2.PEntrez.Pubmed.Pubmed_ResultsPanel.Pubmed_RVAbstractPlus), [von Otte S](http://www.ncbi.nlm.nih.gov/sites/entrez?Db=pubmed&Cmd=Search&Term="von Otte S"%5BAuthor%5D&itool=EntrezSystem2.PEntrez.Pubmed.Pubmed_ResultsPanel.Pubmed_RVAbstractPlus), [Hornung D](http://www.ncbi.nlm.nih.gov/sites/entrez?Db=pubmed&Cmd=Search&Term="Hornung D"%5BAuthor%5D&itool=EntrezSystem2.PEntrez.Pubmed.Pubmed_ResultsPanel.Pubmed_RVAbstractPlus), [Diedrich K](http://www.ncbi.nlm.nih.gov/sites/entrez?Db=pubmed&Cmd=Search&Term="Diedrich K"%5BAuthor%5D&itool=EntrezSystem2.PEntrez.Pubmed.Pubmed_ResultsPanel.Pubmed_RVAbstractPlus). (2005) New developments in the use of peptide gonadotropin-releasing hormone antagonists versus agonists. [*Expert Opin Investig Drugs.*](javascript:AL_get(this, 'jour', 'Expert Opin Investig Drugs.');) 14:1085-97.
8. Shimada K, Ohashi I, Kasahara I, Miyasaka N, Shibuya H. (2004) Triple-phase dynamic MRI of intratumoral vessel density and hyalinization grade in uterine leiomyomas. *AJR Am J Roentgenol* 182:1043-50.
9. Takahashi K, Okada M, Imaoka I, Sugimura K, Miyazaki K. (2001) Value of magnetic resonance imaging in predicting efficacy of GnRH analogue treatment for uterine leiomyoma. *Hum Reprod*. 16:1989-94.
10. Jha RC, Ascher SM, Imaoka I, Spies JB. (2000) Symptomatic fibroleiomyomata: MR imaging of the uterus before and after uterine arterial embolization. *Radiology* 217:228-35
11. Bastin ME, Clayden JD, Pattie A, Gerrish IF, Wardlaw JM, Deary IJ. (2007) Diffusion tensor and magnetization transfer MRI measurements of periventricular white matter hyperintensities in old age. *Neurobiol Aging*: Epub ahead of print.
12. Liapi E, Kamel IR, Bluemke DA, Jacobs MA, Kim HS. (2005) Assessment of response of uterine fibroids and myometrium to embolization using diffusion-weighted echoplanar MR imaging*. J Comput Assist Tomogr* 29(1):83-6.
13. Armitage PA, Schwindack C, Bastin ME, Whittle IR. (2007) Quantitative assessment of intracranial tumor response to dexamethasone using diffusion, perfusion and permeability magnetic resonance imaging. *Magn Reson Imaging* 25:303-10.
14. [**http://www.uk-sh.co.uk/download/1/RADIOLOGY%20Gadolinium%20leaflet.pdf**](http://www.uk-sh.co.uk/download/1/RADIOLOGY Gadolinium leaflet.pdf)
15. [**www.bnf.org**](http://www.bnf.org/)

APPENDIX 1: Summary of Product Characteristics

Cetrotide; <http://www.emea.europa.eu/humandocs/PDFs/EPAR/Cetrotide/H-233-PI-en.pdf>

Zoladex; <http://xpil.medicines.org.uk/FurtherInfo.aspx?DocID=10755>

**CETROTIDE**

**1. NAME OF THE MEDICINAL PRODUCT**

Cetrotide 3 mg powder and solvent for solution for injection

**2. QUALITATIVE AND QUANTITATIVE COMPOSITION**

1 vial contains:

3.12 – 3.24 mg cetrorelix acetate equivalent to 3 mg cetrorelix.

After reconstitution with the solvent provided, the concentration of cetrorelix is 1 mg/ml.

Excipients: 164.40 mg mannitol.

For a full list of excipients, see section 6.1.

**3. PHARMACEUTICAL FORM**

Powder and solvent for solution for injection.

Appearance of the powder: white lyophilized pellet

Appearance of the solvent: clear colourless solution

The pH of the reconstituted solution is 4.0 – 6.0

**4. CLINICAL PARTICULARS**

**4.1 Therapeutic indications**

Prevention of premature ovulation in patients undergoing a controlled ovarian stimulation, followed by oocyte pick-up and assisted reproductive techniques.

In clinical trials Cetrotide 3 mg was used with human menopausal gonadotropin (HMG), however, limited experience with recombinant FSH suggested similar efficacy.

**4.2 Posology and method of administration**

Cetrotide 3 mg should only be prescribed by a specialist experienced in this field.

Cetrotide 3 mg is for subcutaneous injection into the lower abdominal wall.

The first administration of Cetrotide should be performed under the supervision of a physician and under conditions where treatment of possible pseudo-allergic reactions is immediately available. The following injections may be self-administered as long as the patient is made aware of the signs and symptoms that may indicate hypersensitivity, the consequences of such a reaction and the need for immediate medical intervention.

The contents of 1 vial (3 mg cetrorelix) are to be administered on day 7 of ovarian stimulation (approximately 132 to 144 hours after start of ovarian stimulation) with urinary or recombinant gonadotropins. Following the first administration, it is advised that the patient be kept under medical supervision for 30 minutes to ensure there is no allergic/pseudo-allergic reaction reaction to the injection. Facilities for the treatment of such reactions should be immediately available.

If the follicle growth does not allow ovulation induction on the fifth day after injection of Cetrotide 3 mg, additionally 0.25 mg cetrorelix (Cetrotide 0.25 mg) should be administered once daily beginning 96 hours after the injection of Cetrotide 3 mg until the day of ovulation induction.

For instructions on preparation , see section 6.6.

**4.3 Contraindications**

- Hypersensitivity to the active substance or any structural analogues of GnRH, extrinsic peptide hormones or to any of the excipients .
- Pregnancy and lactation.
- Postmenopausal women.
- Patients with moderate and severe renal and hepatic impairment.

**4.4 Special warnings and precautions for use**

Special care should be taken in women with signs and symptoms of active allergic conditions or known history of allergic predisposition. Treatment with Cetrotide is not advised in women with severe allergic conditions.

During or following ovarian stimulation an ovarian hyperstimulation syndrome can occur. This event must be considered as an intrinsic risk of the stimulation procedure with gonadotropins.

An ovarian hyperstimulation syndrome should be treated symptomatically, e.g. with rest, intravenous electrolytes/colloids and heparin therapy.

Luteal phase support should be given according to the reproductive medical centre´s practice.

There is limited experience up to now with the administration of Cetrotide 3 mg during a repeated ovarian stimulation procedure. Therefore Cetrotide 3 mg should be used in repeated cycles only after a careful risk/benefit evaluation.

**4.5 Interaction with other medicinal products and other forms of interaction**

In vitro investigations have shown that interactions are unlikely with medications that are metabolised by cytochrome P450 or glucuronised or conjugated in some other way. However, interactions with commonly used medicinal products, including products that may induce histamine release in susceptible individuals, may occur.

**4.6 Pregnancy and lactation**

Cetrotide 3 mg is not intended to be used during pregnancy and lactation (see section 4.3).

Studies in animals have indicated that cetrorelix exerts a dose related influence on fertility, reproductive performance and pregnancy. No teratogenic effects occurred when the drug was administered during the sensitive phase of gestation.

**4.7 Effects on ability to drive and use machines**

Due to its pharmacological profile cetrorelix is unlikely to impair the patient’s ability to drive or to operate machinery.

**4.8 Undesirable effects**

Within each frequency grouping, undesirable effects are presented in order of decreasing seriousness.

| Immune system disorders | Rare (≥1/10,000, <1/1,000) | Rare cases of hypersensitivity reactions including pseudo-allergic/anaphylactoid reactions have also been reported |
| --- | --- | --- |
| Nervous system disorders | Uncommon (≥1/1,000, <1/100) | Headache |
| Gastrointestinal disorders | Uncommon (≥1/1,000, <1/100) | Nausea |
| Reproductive system and breast disorders | Common (≥1/100, <1/10)  Uncommon (≥1/1,000, <1/100) | Mild to moderate ovarian hyperstimulation syndrome (WHO grade I or II) can occur which is an intrinsic risk of the stimulation procedure (see section 4.4).  Severe ovarian hyperstimulation syndrome (WHO grade III) |
| General disorders and administration site conditions | Common (≥1/100, <1/10) | Local reactions at the injection site (e.g. erythema, swelling and pruritus) have been reported. Usually they were transient in nature and mild intensity. The frequency as reported in clinical trials was 8.0%. |

**4.9 Overdose**

Overdosage in humans may result in a prolonged duration of action but is unlikely to be associated with acute toxic effects.

In acute toxicity studies in rodents non-specific toxic symptoms were observed after intraperitoneal administration of cetrorelix doses more than 200 times higher than the pharmacologically effective dose after subcutaneous administration.

**5 PHARMACOLOGICAL PROPERTIES**

**5.1 Pharmacodynamic properties**

Pharmacotherapeutic group: LHRH-Antagonist, ATC code: H01CC02.

Cetrorelix is a luteinising hormone releasing hormone (LHRH) antagonist. LHRH binds to membrane receptors on pituitary cells. Cetrorelix competes with the binding of endogenous LHRH to these receptors. Due to this mode of action, cetrorelix controls the secretion of gonadotropins (LH and FSH).

Cetrorelix dose-dependently inhibits the secretion of LH and FSH from the pituitary gland. The onset of suppression is virtually immediate and is maintained by continuous treatment, without initial stimulatory effect.

In females, cetrorelix delays the LH surge and consequently ovulation.

In women undergoing ovarian stimulation the duration of action of cetrorelix is dose dependent. Following a single dose of 3 mg of cetrorelix a duration of action of at least 4 days has been evaluated. On day 4 the suppression was approximately 70%. At a dose of 0.25 mg per injection repeated injections every 24 hours will maintain the effect of cetrorelix.

In animals as well as in humans, the antagonistic hormonal effects of cetrorelix were fully reversible after termination of treatment.

**5.2 Pharmacokinetic properties**

The absolute bioavailability of cetrorelix after subcutaneous administration is about 85%.

The total plasma clearance and the renal clearance are 1.2 ml x min^-1^ x kg^-1^ and

0.1 ml x min^-1^ x kg^-1^, respectively. The volume of distribution (V_d,area_) is 1.1 l x kg^-1^. The mean terminal half-lives following intravenous and subcutaneous administration are about 12 h and 30 h, respectively, demonstrating the effect of absorption processes at the injection site. The subcutaneous administration of single doses (0.25 mg to 3 mg cetrorelix) and also daily dosing over 14 days show linear kinetics.

**5.3 Preclinical safety data**

Non-clinical data reveal no special hazard for humans based on conventional studies of safety pharmacology, repeated dose toxicity, genotoxicity, carcinogenic potential, toxicity to reproduction.

No target organ toxicity could be observed from acute, subacute and chronic toxicity studies in rats and dogs following subcutaneous administration of cetrorelix. No signs of drug-related local irritation or incompatibility were noted in dogs after intravenous, intra-arterial and paravenous injection when cetrorelix was administered in doses clearly above the intended clinical use in man.

Cetrorelix showed no mutagenic or clastogenic potential in gene and chromosome mutation assays.

**6. PHARMACEUTICAL PARTICULARS**

**6.1 List of excipients**

Powder:

Mannitol

Solvent:

Water for Injections

**6.2 Incompatibilities**

This medicinal product must not be mixed with other medicinal products except those mentioned in section 6.6

**6.3 Shelf life**

2 years.

The solution should be used immediately after preparation.

**6.4 Special precautions for storage**

Do not store above 25 °C. Keep the vial(s) in the outer carton in order to protect from light.

**6.5 Nature and contents of container**

Packs with 1 Type I glass vial containing 167.7 mg powder for solution for injection sealed with a rubber stopper.

Additionally the pack contains:

1 pre-filled syringe (Type I glass cartridge closed with rubber stoppers) with 3 ml solvent for parenteral use

1 injection needle (20 gauge)

1 hypodermic injection needle (27 gauge)

2 alcohol swabs.

**6.6 Special precautions for disposal and other handling**

Cetrotide 3 mg should only be reconstituted with the solvent provided, using a gentle, swirling motion. Vigorous shaking with bubble formation should be avoided.

The reconstituted solution is without particles and clear. Do not use if the solution contains particles or if the solution is not clear.

Withdraw the entire contents of the vial. This ensures a delivery to the patient of a dose of at least 2.82 mg cetrorelix.

The solution should be used immediately after reconstitution.

The injection site should be varied daily.

**7. MARKETING AUTHORISATION HOLDER**

Serono Europe Limited

56 Marsh Wall

London E14 9TP

United Kingdom

**8. MARKETING AUTHORISATION NUMBER(S)**

EU/1/99/100/003

**9. DATE OF FIRST AUTHORISATION / RENEWAL OF THE AUTHORISATION**

13 April 1999

Date of last renewal: 15 April 2004

**10. DATE OF REVISION OF THE TEXT**

ZOLADEX

**1. NAME OF THE MEDICINAL PRODUCT**

Zoladex®3.6 mg Implant

**2. QUALITATIVE AND QUANTITATIVE COMPOSITION**

Goserelin acetate (equivalent to 3.6 mg goserelin).

For excipients, see 6.1.

**3. PHARMACEUTICAL FORM**

Implant, in pre-filled syringe.

**4. CLINICAL PARTICULARS**

**4.1 Therapeutic indications**

(i) Treatment of prostate cancer in the following settings (see also section 5.1):

- In the treatment of metastatic prostate cancer
- In the treatment of locally advanced prostate cancer, as an alternative to surgical castration
- As adjuvant treatment to radiotherapy in patients with high-risk localised or locally advanced prostate cancer
- As neo-adjuvant treatment prior to radiotherapy in patients with high-risk localised or locally advanced prostate cancer
- As adjuvant treatment to radical prostatectomy in patients with locally advanced prostate cancer at high risk of disease progression

(ii) Advanced breast cancer in pre and perimenopausal women suitable for hormonal manipulation.

(iii) Zoladex 3.6 mg is indicated as an alternative to chemotherapy in the standard of care for pre/perimenopausal women with oestrogen receptor (ER) positive early breast cancer.

(iv) Endometriosis: In the management of endometriosis, Zoladex alleviates symptoms, including pain, and reduces the size and number of endometrial lesions.

(v) Endometrial thinning: Zoladex is indicated for the prethinning of the uterine endometrium prior to endometrial ablation or resection.

(vi) Uterine fibroids: In conjunction with iron therapy in the haematological improvement of anaemic patients with fibroids prior to surgery.

(vii) Assisted reproduction: Pituitary downregulation in preparation for superovulation.

**4.2 Posology and method of administration**

**Adults**

One 3.6 mg depot of Zoladex injected subcutaneously into the anterior abdominal wall, every 28 days. No dosage adjustment is necessary for patients with renal or hepatic impairment, or in the elderly.

Endometriosis should be treated for a period of six months only, since at present there are no clinical data for longer treatment periods. Repeat courses should not be given due to concern about loss of bone mineral density. In patients receiving Zoladex for the treatment of endometriosis, the addition of hormone replacement therapy (a daily oestrogenic agent and a progestogenic agent) has been shown to reduce bone mineral density loss and vasomotor symptoms.

For use in endometrial thinning: four or eight weeks treatment. The second depot may be required for the patient with a large uterus or to allow flexible surgical timing.

For women who are anaemic as a result of uterine fibroids: Zoladex 3.6 mg depot with supplementary iron may be administered for up to three months before surgery.

Assisted reproduction: Zoladex 3.6 mg is administered to downregulate the pituitary gland, as defined by serum estradiol levels similar to those observed in the early follicular phase (approximately 150 pmol/l). This will usually take between 7 and 21 days.

When downregulation is achieved, superovulation (controlled ovarian stimulation) with gonadotrophin is commenced. The downregulation achieved with a depot agonist is more consistent suggesting that, in some cases, there may be an increased requirement for gonadotrophin. At the appropriate stage of follicular development, gonadotrophin is stopped and human chorionic gonadotrophin (hCG) is administered to induce ovulation. Treatment monitoring, oocyte retrieval and fertilisation techniques are performed according to the normal practice of the individual clinic.

**Children**

Zoladex is not indicated for use in children.

For correct administration of Zoladex, see instructions on the instruction card.

**4.3 Contraindications**

Zoladex should not be given to patients with a known hypersensitivity to the active substance, to other LHRH analogues, or to any of the excipients of this product.

Zoladex should not be used in pregnancy (see section 4.6).

**4.4 Special warnings and precautions for use**

Zoladex is not indicated for use in children, as safety and efficacy have not been established in this group of patients.

**Males**

The use of Zoladex in men at particular risk of developing ureteric obstruction or spinal cord compression should be considered carefully, and the patients monitored closely during the first month of therapy. Consideration should be given to the initial use of an anti-androgen (e.g. cyproterone acetate 300 mg daily for three days before and three weeks after commencement of Zoladex) at the start of LHRH analogue therapy since this has been reported to prevent the possible sequelae of the initial rise in serum testosterone. If spinal cord compression or renal impairment due to ureteric obstruction are present or develop, specific standard treatment of these complications should be instituted.

**Females**

The use of LHRH agonists in women may cause a loss of bone mineral density. Following two years treatment for early breast cancer, the average loss of bone mineral density was 6.2% and 11.5% at the femoral neck and lumbar spine respectively. This loss has been shown to be partially reversible at the one year off treatment follow-up with recovery to 3.4% and 6.4% relative to baseline at the femoral neck and lumbar spine respectively, although this recovery is based on very limited data.

In patients receiving Zoladex for the treatment of endometriosis, the addition of hormone replacement therapy (a daily oestrogenic agent and a progestogenic agent), has been shown to reduce bone mineral density loss and vasomotor symptoms.

Zoladex should be used with caution in women with known metabolic bone disease.

Zoladex may cause an increase in uterine cervical resistance, which may result in difficulty in dilating the cervix.

Currently, there are no clinical data on the effect of treating benign gynaecological conditions with Zoladex for periods in excess of six months.

Zoladex should only be administered as part of a regimen for assisted reproduction under the supervision of a specialist experienced in the area.

As with other LHRH agonists, there have been reports of ovarian hyperstimulation syndrome (OHSS), associated with the use of Zoladex 3.6 mg in combination with gonadotrophin. It has been suggested that the downregulation achieved with a depot agonist may lead, in some cases, to an increased requirement for gonadotrophin. The stimulation cycle should be monitored carefully to identify patients at risk of developing OHSS because its severity and incidence may be dependent on the dose regimen of gonadotrophin. Human chorionic gonadotrophin (hCG) should be withheld, if appropriate.

It is recommended that Zoladex is used with caution in assisted reproduction regimens in patients with polycystic ovarian syndrome as follicle recruitment may be increased.

**4.5 Interaction with other medicinal products and other forms of interaction**

None known.

**4.6 Pregnancy and lactation**

Pregnancy: Although reproductive toxicity in animals gave no evidence of teratogenic potential, Zoladex should not be used in pregnancy as there is a theoretical risk of abortion or foetal abnormality if LHRH agonists are used during pregnancy. Potentially fertile women should be examined carefully before treatment to exclude pregnancy. Non-hormonal methods of contraception should be employed during therapy and in the case of endometriosis should be continued until menses are resumed.

Pregnancy should be excluded before Zoladex is used for assisted reproduction. The clinical data from use in this setting are limited but the available evidence suggests there is no causal association between Zoladex and any subsequent abnormalities of oocyte development or pregnancy and outcome.

Lactation: The use of Zoladex during breast-feeding is not recommended.

**4.7 Effects on ability to drive and use machines**

There is no evidence that Zoladex results in impairment of these activities.

**4.8 Undesirable effects**

**General**

Rare incidences of hypersensitivity reactions, which may include some manifestations of anaphylaxis, have been reported.

Arthralgia has been reported. Non-specific paraesthesias have been reported. Skin rashes have been reported which are generally mild, often regressing without discontinuation of therapy.

Changes in blood pressure, manifest as hypotension or hypertension, have been occasionally observed in patients administered Zoladex. The changes are usually transient, resolving either during continued therapy or after cessation of therapy with Zoladex. Rarely, such changes have been sufficient to require medical intervention including withdrawal of treatment from Zoladex.

As with other agents in this class, very rare cases of pituitary apoplexy have been reported following initial administration.

Occasional local reactions include mild bruising at the subcutaneous injection site.

**Males**

Pharmacological effects in men include hot flushes and sweating and a decrease in libido, seldom requiring withdrawal of therapy. Breast swelling and tenderness have been noted infrequently. Initially, prostate cancer patients may experience a temporary increase in bone pain, which can be managed symptomatically. Isolated cases of ureteric obstruction and spinal cord compression have been recorded.

The use of LHRH agonists in men may cause a loss of bone mineral density.

**Females**

Pharmacological effects in women include hot flushes and sweating, and loss in libido, seldom requiring withdrawal of therapy. Headaches, mood changes including depression, vaginal dryness and change in breast size have been noted. During early treatment with Zoladex some women may experience vaginal bleeding of variable duration and intensity. If vaginal bleeding occurs it is usually in the first month after starting treatment. Such bleeding probably represents oestrogen withdrawal bleeding and is expected to stop spontaneously.

Initially, breast cancer patients may experience a temporary increase in signs and symptoms, which can be managed symptomatically. In women with fibroids, degeneration of fibroids may occur.

Rarely, breast cancer patients with metastases have developed hypercalcaemia on initiation of therapy.

Rarely, some women may enter the menopause during treatment with LHRH analogues and not resume menses on cessation of therapy. This may simply be a physiological change.

In assisted reproduction: As with other LHRH agonists, there have been reports of ovarian hyperstimulation syndrome (OHSS), associated with the use of Zoladex 3.6 mg in combination with gonadotrophin. It has been suggested that the downregulation achieved with a depot agonist may lead, in some cases, to an increased requirement for gonadotrophin. The stimulation cycle should be monitored carefully to identify patients at risk of developing OHSS because its severity and incidence may be dependent on the dose regimen of gonadotrophin. Human chorionic gonadotrophin (hCG) should be withheld, if appropriate.

Follicular and luteal ovarian cysts have been reported to occur following LHRH therapy. Most cysts are asymptomatic, non functional, varying in size and resolve spontaneously.

**4.9 Overdose**

There is limited experience of overdosage in humans. In cases where Zoladex has unintentionally been re-administered early or given at a higher dose, no clinically relevant adverse effects have been seen. Animal tests suggest that no effect other than the intended therapeutic effects on sex hormone concentrations and on the reproductive tract will be evident with higher doses of Zoladex. If overdosage occurs, this should be managed symptomatically.

**5 PHARMACOLOGICAL PROPERTIES**

**5.1 Pharmacodynamic properties**

Zoladex (D-Ser(Bu^t^)^6^ Azgly^10^ LHRH) is a synthetic analogue of naturally occurring LHRH. On chronic administration Zoladex results in inhibition of pituitary LH secretion leading to a fall in serum testosterone concentrations in males and serum estradiol concentrations in females. This effect is reversible on discontinuation of therapy. Initially, Zoladex, like other LHRH agonists, may transiently increase serum testosterone concentration in men and serum estradiol concentration in women.

In men, by around 21 days after the first depot injection, testosterone concentrations have fallen to within the castrate range and remain suppressed with continuous treatment every 28 days. This inhibition leads to prostate tumour regression and symptomatic improvement in the majority of patients.

In the management of patients with metastatic prostate cancer, Zoladex has been shown in comparative clinical trials to give similar survival outcomes to those obtained with surgical castrations.

In comparative trials, Zoladex has been shown to improve disease-free survival and overall survival when used as an adjuvant therapy to radiotherapy in patients with high-risk localised (T_1_-T_2_ and PSA of at least 10 ng/mL or a Gleason score of at least 7), or locally advanced (T_3_-T_4_) prostate cancer. The optimum duration of adjuvant therapy has not been established; a comparative trial has shown that 3 years of adjuvant Zoladex gives significant survival improvement compared with radiotherapy alone. Neo-adjuvant Zoladex prior to radiotherapy has been shown to improve disease-free survival in patients with high risk localised or locally advanced prostate cancer.

After prostatectomy, in patients found to have extra-prostatic tumour spread, adjuvant Zoladex may improve disease-free survival periods, but there is no significant survival improvement unless patients have evidence of nodal involvement at time of surgery. Patients with pathologically staged locally advanced disease should have additional risk factors such as PSA of at least 10 ng/mL or a Gleason score of at least 7 before adjuvant Zoladex should be considered. There is no evidence of improved clinical outcomes with use of neo-adjuvant Zoladex before radical prostatectomy.

In women, serum estradiol concentrations are suppressed by around 21 days after the first depot injection and, with continuous treatment every 28 days, remain suppressed at levels comparable with those observed in postmenopausal women. This suppression is associated with a response in hormone-dependent advanced breast cancer**,** uterine fibroids, endometriosis and suppression of follicular development within the ovary. It will produce endometrial thinning and will result in amenorrhoea in the majority of patients.

During treatment with LHRH analogues patients may enter the menopause. Rarely, some women do not resume menses on cessation of therapy.

Zoladex in combination with iron has been shown to induce amenorrhoea and improve haemoglobin concentrations and related haematological parameters in women with fibroids who are anaemic. The combination produced a mean haemoglobin concentration 1 g/dl above that achieved by iron therapy alone.

**5.2 Pharmacokinetic properties**

The bioavailability of Zoladex is almost complete. Administration of a depot every four weeks ensures that effective concentrations are maintained with no tissue accumulations. Zoladex is poorly protein bound and has a serum elimination half-life of two to four hours in subjects with normal renal function. The half-life is increased in patients with impaired renal function. For the compound given monthly in a depot formulation, this change will have minimal effect. Hence, no change in dosing is necessary in these patients. There is no significant change in pharmacokinetics in patients with hepatic failure.

**5.3 Preclinical safety data**

Following long-term repeated dosing with Zoladex, an increased incidence of benign pituitary tumours has been observed in male rats. Whilst this finding is similar to that previously noted in this species following surgical castration, any relevance to man has not been established.

In mice, long-term repeated dosing with multiples of the human dose, produced histological changes in some regions of the digestive system manifested by pancreatic islet cell hyperplasia and a benign proliferative condition in the pyloric region of the stomach, also reported as a spontaneous lesion in this species. The clinical relevance of these findings is unknown.

**6. PHARMACEUTICAL PARTICULARS**

**6.1 List of excipients**

Lactide/glycolide copolymer.

**6.2 Incompatibilities**

None known.

**6.3 Shelf life**

36 months.

The solution should be used immediately after preparation.

**6.4 Special precautions for storage**

Do not store above 25 °C.

**6.5 Nature and contents of container**

Single dose Safe System™ syringe applicator with a protective sleeve.

**6.6 Special precautions for disposal and other handling**

Use as directed by the prescriber. Use only if pouch is undamaged. Use immediately after opening pouch. Dispose of the syringe in an approved sharps collector.

**7. MARKETING AUTHORISATION HOLDER**

AstraZeneca UK Limited,

600 Capability Green,

Luton, LU1 3LU, UK.

**8. MARKETING AUTHORISATION NUMBER(S)**

PL 17901/0064

**9. DATE OF FIRST AUTHORISATION / RENEWAL OF THE AUTHORISATION**

1^st^ May 2001 (formerly 13.05.1993)

**10. DATE OF REVISION OF THE TEXT**

<http://emc.medicines.org.uk/emc/assets/c/html/DisplayDoc.asp?DocumentID=7855>

SUPPORTING INFORMATION

Patient Information Leaflet:
[Zoladex 3.6mg Implant](http://emc.medicines.org.uk/emc/assets/c/html/DisplayDoc.asp?DocumentID=10755)

Alternative format PIL:
[Zoladex 3.6mg Implant (new window)](http://xpil.medicines.org.uk/ViewPil.aspx?docid=10755)

Medicine Guide:
[Zoladex (new window)](http://medguides.medicines.org.uk/document.aspx?name=Zoladex)

APPENDIX 2: Monitoring Plan

1. Trial oversight committees:

|  | **Y** | **N** | **N/A** |
| --- | --- | --- | --- |
| Trial management group |  | X |  |
| Independent data monitoring committee |  | X |  |
| Trial Steering committee |  | X |  |
| Other/comments: | | | |

2. Central monitoring:

|  | **Y** | **N** | **N/A** |
| --- | --- | --- | --- |
| Data collected and validated via central coordinating centre (‘good housekeeping’) |  | X |  |
| Trial data recorded on database with inbuilt data checks (eg value ranges etc) |  | X |  |
| Randomisation done via central coordinating centre (verify eligibility) |  | X |  |
| Central pharmacovigilance reporting procedure | X |  |  |
| Investigator training/Investigator meetings |  |  | X  (single site) |
| Other/comments: | | | |

3. On-site monitoring:

|  | **Y** | **N** | **N/A** |
| --- | --- | --- | --- |
| Investigator Site File | X |  |  |
| SDV informed consent | X |  |  |
| SDV of critical data (eg endpoint data, dose adjustments, concomitant medication) | X |  |  |
| SDV of safety data (AEs, SAE, SUSARs) and timeliness of reporting | X |  |  |
| SDV patient eligibility | X |  |  |
| Storage and security of trial data | X |  |  |
| Pharmacy documentation |  | X |  |
| Study drug accountability |  | X |  |
| Study drug storage |  | X |  |
| Other/comments:  IMP used within licence therefore pharmacy will not be monitored unless requested by CI/Co-Sponsor | | | |

**4. Expected frequency of on-site monitoring of lead site (or local site) and document the plan for monitoring of external sites if applicable:**

(This will be based on the risk assessment and the number and type of central monitoring strategies for a particular trial).

| This is a single site study.  Study start-up visit prior to recruitment.  1-2 visits during patient recruitment to review ISF, SDV trial data, check 100% consent forms.  Study closure visit after patient recruitment and analysis of results. |
| --- |

APPENDIX 3: Risk Assessment

**RISK ASSESSMENT TOOL FOR MONITORING**

| **MONITORING CRITERIA*** | **Y** | **N** | **N/A** |
| --- | --- | --- | --- |
| Is the research sponsored or co-sponsored by The University of Edinburgh and/or NHS Lothian? | X |  |  |
| IMP trial?   - IMP used in novel setting or indication not listed in SmPC - IMP to be used within strict settings? (e.g. storage temperatures, short shelf life etc). | X | X  X |  |
| Hazards of assessment methods eg x-ray, biopsy etc |  | X |  |
| Risk of SAE/SUSARs (greater than that expected as part of routine care)? |  | X |  |
| Research involving children?  Research involving other vulnerable patient group? |  | X  X |  |
| Complex protocol? |  | X |  |
| Large intake? |  | X |  |
| Randomised or blinded trial? | X |  |  |
| Potential for high subject drop-out/withdrawal rate? |  | X |  |

*Studies will be monitored if they meet one or more of the above criteria. Studies that meet multiple criteria will be monitored in more depth and more frequently than studies that meet only one criterion.

APPENDIX 4: Study plan

Pilot Group A - GnRH agonist Only (Goserelin 3.6mgs) (n=15)

| **Day of Treatment** | **Visits/Interventions** | **U&Es**  **FBC**  **LFTs** | **Concomitant**  **Meds** | **Adverse Events** | **E2, P, FSH, LH** |
| --- | --- | --- | --- | --- | --- |
| Screening | Informed Consent  Inclusion & Exclusion Criteria  Medical & Menstrual History  Physical Exam & Vital Signs  Height & Weight  Urinalysis & Pregnancy Test  Ultrasound  Doppler (if required) | **X** | **X** |  |  |
| Within  28 days  of Day 1 | **Visit 1**  1.5T MRI, Ultrasound & Doppler (if required)  Cannulation & Gadolinium |  | **X** | **X** |  |
|  | **GnRH agonist**  (Goserelin 3.6mg s/c)  **clinically indicated and requested by clinician** |  |  |  |  |
| **1**  (Days 1 to 5 of cycle) | GnRH agonist **Dose 1**  (Goserelin 3.6mg s/c) |  | **X** | **X** |  |
| 28 days from Dose 1 +/- 3 days | GnRH agonist **Dose 2**  (Goserelin 3.6mg s/c) | **X** | **X** | **X** |  |
| 28 days from Dose 2 +/- 3 days | GnRH agonist **Dose 3**  (Goserelin 3.6mg s/c)  This dose may not be  clinically requested |  | **X** | **X** |  |
| Up to 10 days  Prior to  Hysterectomy | **Visit 2**  1.5T MRI, Ultrasound & Doppler  Cannulation & Gadolinium |  | **X** | **X** |  |
| 21 – 28 days from  GnRH agonist Dose 2 or 3 | **Visit 3**  Hysterectomy  7T MRI of Uterus  Histo-morphology | **X** | **X** | **X** | **X** |

(Version 2 13th October 2009)

**The following are NOT required for Pilot Groups -**

**Urine Sample (5mls) collected twice weekly from Visit 1**

**Daily Menstrual Recording Chart from Visit 1**

**Pilot Group B – No treatment (n=1**5)

| **Day of Treatment** | **Visits/Interventions** | **U&Es**  **FBC**  **LFTs** | **Concomitant**  **Meds** | **Adverse Events** | **E2, P, FSH, LH** |
| --- | --- | --- | --- | --- | --- |
| Screening | Informed Consent  Inclusion & Exclusion Criteria  Medical & Menstrual History  Physical Exam & Vital Signs  Height & Weight  Urinalysis & Pregnancy Test  Ultrasound  Doppler (if required) | **X** | **X** |  |  |
|  | **No treatment requested by clinician** |  |  |  |  |
| **Up to 10 days**  **Prior to**  **Hysterectomy** | **Visit 1**  1.5T MRI, Ultrasound & Doppler (if required)  Cannulation & Gadolinium |  | **X** | **X** |  |
|  | **Visit 2**  Hysterectomy  7T MRI of Uterus  Histo-morphology | **X** | **X** | **X** | **X** |

(Version 2 13^th^ October 2009)

**The following are NOT required for Pilot Groups -**

**Urine Sample (5mls) collected twice weekly from Visit 1**

**Daily Menstrual Recording Chart from Visit 1**

**Pilot Group C – No treatment 2 Novel MRI scans (n=5)**

| **Day of Treatment** | **Visits/Interventions** | **U&Es**  **FBC**  **LFTs** | **Concomitant**  **Meds** | **Adverse Events** | **E2, P, FSH, LH** |
| --- | --- | --- | --- | --- | --- |
| Screening | Informed Consent  Inclusion & Exclusion Criteria  Medical & Menstrual History  Physical Exam & Vital Signs  Height & Weight  Urinalysis & Pregnancy Test  Ultrasound  Doppler (if required) | **X** | **X** |  |  |
|  | **No treatment requested by clinician** |  |  |  |  |
| **Up to 10 days**  **Prior to**  **Hysterectomy** | **Visit 1**  1.5T MRI, 3T MRI, Ultrasound & Doppler (if required)  Cannulation & Gadolinium |  | **X** | **X** |  |
|  | **Visit 2**  Hysterectomy  7T MRI of Uterus  Histo-morphology | **X** | **X** | **X** | **X** |

(Version 1 13^th^ October 2009)

**The following are NOT required for Pilot Groups -**

**Urine Sample (5mls) collected twice weekly from Visit 1**

**Daily Menstrual Recording Chart from Visit 1**

**Group 1 GnRH agonist Only (Goserelin 3.6mgs) (n=10)**

| **Day of Treatment** | **Visits/Interventions** | **U&Es**  **FBC**  **LFTs** | **E2, P4,**  **LH, FSH**  **Time Points** | **Concomitant**  **Meds** | **Adverse Events** |
| --- | --- | --- | --- | --- | --- |
| Screening | Informed Consent  Inclusion & Exclusion Criteria  Medical & Menstrual History  Physical Exam & Vital Signs  Height & Weight  Urinalysis & Pregnancy Test  Ultrasound  Doppler (if required**)** | **X** |  | **X** |  |
| Within  28 days  of Day 1 | **Visit 1**  1.5T MRI, Ultrasound & Doppler (if required)  Cannulation & Gadolinium |  | **1** | **X** | **X** |
|  | **Randomisation** |  |  |  |  |
| **1**  (Days 1 to 5 of cycle) | GnRH agonist **Dose 1**  (Goserelin 3.6mg s/c) |  | **2** | **X** | **X** |
| 4+/- 1 |  |  | **3** | **X** | **X** |
| 7+/- 1 |  |  | **4** | **X** | **X** |
| 11+/- 1 |  |  | **5** | **X** | **X** |
| 14+/- 3 | **Visit 2**  1.5T MRI, Ultrasound & Doppler |  | **6** | **X** | **X** |
| 28 +/- 3 days from Dose 1 | **Visit 3**  GnRH agonist **Dose 2**  (Goserelin 3.6mg s/c)  MRI, Ultrasound & Doppler  Cannulation & Gadolinium | **X** | **7** | **X** | **X** |
| 28 +/- 3 days from Dose 2 | GnRH agonist **Dose 3**  (Goserelin 3.6mg s/c) |  | **8** | **X** | **X** |
| Within 10 days of TAH | **Visit 4**  1.5T MRI, Ultrasound & Doppler  Cannulation & Gadolinium |  | **9** | **X** | **X** |
| 21-28 days from Dose 3 | **Visit 5**  Hysterectomy  7T MRI of Uterus  Histo-morphology | **X** | **10** | **X** | **X** |

(Version 2 13^th^ October 2009)

**In Addition -**

**Urine Sample (5mls) collected twice weekly from Visit 1**

**Daily Menstrual Recording Chart from Visit 1**

**Group 2 GnRH antagonist (Cetrorelix 3mgs) and GnRH agonist (Goserelin** 3.6mgs)(n=10)

| **Day of Treatment** | **Visits/Interventions** | **U&Es**  **FBC**  **LFTs** | **E2, P4,**  **LH, FSH**  **Time Points** | **Concomitant**  **Meds** | **Adverse Events** |
| --- | --- | --- | --- | --- | --- |
| Screening | Informed Consent  Inclusion & Exclusion Criteria  Medical & Menstrual History  Physical Exam & Vital Signs  Height & Weight  Urinalysis & Pregnancy Test  Ultrasound  Doppler (if required) | **X** |  | **X** |  |
| Within  28 days  of Day 1 | **Visit 1**  1.5T MRI, Ultrasound & Doppler (if required**)**  Cannulation & Gadolinium |  | **1** | **X** | **X** |
|  | **Randomisation** |  |  |  |  |
| 1  (Days 1 to 5 of cycle) | GnRH antagonist **Dose 1**  (3 mg Cetrorelix s/c) |  | **2** | **X** | **X** |
| 4+/- 1 day | GnRH antagonist **Dose 2**  (3 mg Cetrorelix s/c) |  | **3** | **X** | **X** |
| 7+/- 1 day | GnRH antagonist **Dose 3**  (3 mg Cetrorelix s/c)  GnRH agonist **Dose 1**  (Goserelin 3.6mg s/c) |  | **4** | **X** | **X** |
| 11+/- 1 day |  |  | **5** | **X** | **X** |
| 14+/- 3 days | **Visit 2**  1.5T MRI, Ultrasound & Doppler  Cannulation & Gadolinium |  | **6** | **X** | **X** |
| 28+/- 3 days | **Visit 3**  1.5T MRI, Ultrasound & Doppler  Cannulation & Gadolinium | **X** | **7** | **X** | **X** |
| 28 +/- 3 days from Dose 1 Goserelin | GnRH agonist **Dose 2**  (Goserelin 3.6mg s/c) |  | **7A** | **X** | **X** |
| 28 +/- 3 days from Dose 2 Goserelin | GnRH agonist **Dose 3**  (Goserelin 3.6mg s/c) |  | **8** | **X** | **X** |
| Within 10 days of TAH | **Visit 4**  1.5T MRI, Ultrasound & Doppler  Cannulation & Gadolinium |  | **9** | **X** | **X** |
| 21-28 days from Dose 3 Goserelin | **Visit 5**  Hysterectomy  7T MRI of Uterus  Histo-morphology | **X** | **10** | **X** | **X** |

(Version 2 13^th^ October 2009)

**In Addition -**

**Urine Sample (5mls) collected twice weekly from Visit 1**

**Daily Menstrual Recording Chart from Visit 1**

**Control – No Medication (n=10)**

| **Day of Treatment** | **Visits/Interventions** | **U&Es**  **FBC**  **LFTs** | **E2, P4,**  **LH, FSH**  **Time Points** | **Concomitant**  **Meds** | **Adverse Events** |
| --- | --- | --- | --- | --- | --- |
| Screening | Informed Consent  Inclusion & Exclusion Criteria  Medical & Menstrual History  Physical Exam & Vital Signs  Height & Weight  Urinalysis & Pregnancy Test  Ultrasound  Doppler (if required) | **X** |  | **X** |  |
| Within  28 days  of Day 1 | **Visit 1**  1.5T MRI, Ultrasound & Doppler (if required)  Cannulation & Gadolinium |  | **1** | **X** | **X** |
|  | **Randomisation** |  |  |  |  |
| 1  (Days 1 to 5 of cycle) | No Medication |  | **2** | **X** | **X** |
| 4+/- 1 day |  |  | **3** | **X** | **X** |
| 7+/- 1 day |  |  | **4** | **X** | **X** |
| 11+/- 1 day |  |  | **5** | **X** | **X** |
| 14+/-3 days | **Visit 2**  1.5T MRI, Ultrasound & Doppler  Cannulation & Gadolinium |  | **6** | **X** | **X** |
| 28+/-3 days from Visit 2 | **Visit 3**  1.5T MRI, Ultrasound & Doppler  Cannulation & Gadolinium | **X** | **7** | **X** | **X** |
| 28+/- 3 days from Visit 3 |  |  | **8** | **X** | **X** |
| Within 10 days of TAH | **Visit 4**  1.5T MRI, Ultrasound & Doppler  Cannulation & Gadolinium |  | **9** | **X** | **X** |
| 21-28 days from Visit 3 | **Visit 5**  Hysterectomy  7T MRI of Uterus  Histo-morphology | **X** | **10** | **X** | **X** |

(Version 2 13^th^ October 2009)

**In Addition -**

**Urine Sample (5mls) collected twice weekly from Visit 1**

**Daily Menstrual Recording Chart from Visit 1**
